# Comparative Effects of Event Detection Methods on the Analysis and Interpretation of Ca^2+^ Imaging Data

**DOI:** 10.3389/fnins.2021.620869

**Published:** 2021-03-26

**Authors:** Austin Neugornet, Bernadette O’Donovan, Pavel Ivanovich Ortinski

**Affiliations:** ^1^Department of Neuroscience, School of Medicine, University of Kentucky, Lexington, KY, United States; ^2^Department of Pharmacology, Physiology and Neuroscience, University of South Carolina School of Medicine, Columbia, SC, United States

**Keywords:** calcium imaging, event identification, network analysis, astrocytes, calcium transient detection

## Abstract

Calcium imaging has gained substantial popularity as a tool to profile the activity of multiple simultaneously active cells at high spatiotemporal resolution. Among the diverse approaches to processing of Ca^2+^ imaging data is an often subjective decision of how to quantify baseline fluorescence or *F*_0_. We examine the effect of popular *F*_0_ determination methods on the interpretation of neuronal and astrocyte activity in a single dataset of rats trained to self-administer intravenous infusions of cocaine and compare them with an *F*_0_-independent wavelet ridgewalking event detection approach. We find that the choice of the processing method has a profound impact on the interpretation of widefield imaging results. All of the d*F*/*F*_0_ thresholding methods tended to introduce spurious events and fragment individual transients, leading to smaller calculated event durations and larger event frequencies. Analysis of simulated datasets confirmed these observations and indicated substantial intermethod variability as to the events classified as significant. Additionally, most d*F*/*F*_0_ methods on their own were unable to adequately account for bleaching of fluorescence, although the *F*_0_ smooth approach and the wavelet ridgewalking algorithm both did so. In general, the choice of the processing method led to dramatically different quantitative and sometimes opposing qualitative interpretations of the effects of cocaine self-administration both at the level of individual cells and at the level of cell networks. Significantly different distributions of event duration, amplitude, frequency, and network measures were found across the majority of d*F*/*F*_0_ approaches. The wavelet ridgewalking algorithm broadly outperformed d*F*/*F*_0_-based methods for both neuron and astrocyte recordings. These results indicate the need for heightened awareness of the limitations and tendencies associated with decisions to use particular Ca^2+^ image processing pipelines. Both quantification and interpretation of the effects of experimental manipulations are strongly sensitive to such decisions.

## Introduction

Development of genetically encoded calcium indicators (GECIs) has encouraged a bloom of research to capture the activity of large cell populations at high spatiotemporal resolution. The activity of diverse cell types can be examined using Ca^2+^ imaging approaches. For example, amplitude and duration of neuronal Ca^2+^ transients are typically interpreted as proxies for action potentials (Ali and Kwan, 2020), and diverse features of astrocytic Ca^2+^ have been proposed to play functional roles in neural circuit regulation, behavior, and information processing (Guerra-Gomes et al., 2017). Accurate identification of Ca^2+^ transients is critical to facilitate understanding of dynamic behavior in individual cells as well as circuit relationships that may arise from cell–cell interactions. A variety of analytical approaches to process large amounts of imaging data are available, but the extent to which the choice of the analysis pipeline may impact interpretation of the underlying data remains unclear.

Most “traditional” event identification methods are based on transforming a fluorescence signal into d*F*/*F*_0_ and then applying a threshold to find significant deviations from the presumed background noise. Definitions of baseline *F*_0_ and thresholding methods vary widely across studies. In our analyses, we implement three common *F*_0_ definitions: an initial segment of the fluorescence trace (*F*_0_ initial), often used in recordings involving extracellular stimulations (e.g., Ellefsen et al., 2014; Lock et al., 2015; Rahmati et al., 2016); a minimally variable and dim segment (*F*_0_ minimal); and as a fit to the background in a sliding window throughout the trace (*F*_0_ smooth), such as implemented in a toolbox by Romano et al. (2017), SICT (Mancini et al., 2018), or FluoroSNNAP (Patel et al., 2015).

Wavelet ridgewalking algorithms, as originally developed for use in mass spectroscopy signal analysis (Du et al., 2006), have been recently employed to find events in neuronal imaging data (Prada et al., 2018). Wavelet ridgewalking algorithms make minimal assumptions about the characteristics of events they identify, requiring only that they are “peak-like” at some scale. Events across a wide range of durations and amplitude scales can be identified, even in the presence of high levels of noise. The wavelet method lacks precise event shape, duration, or amplitude requirements, increasing its versatility and potential to identify morphologically diverse Ca^2+^ waveforms. This is of particular importance for processing astrocytic data, as these cells are known to exhibit highly heterogeneous Ca^2+^ signals (Shigetomi et al., 2016; Verkhratsky and Nedergaard, 2018).

As Ca^2+^ imaging recordings capture activity within a broad local network, they can be used to explore network structure under a variety of paradigms. Graph theory has been developed to quantify and examine network characteristics, describing the organization of groups of objects of interest (nodes) through their connections to each other (edges) (Bullmore and Sporns, 2009). Graph theory is currently finding extensive use throughout neuroscience, with particularly prominent applications in neuroimaging and clinical psychiatry (Rubinov and Sporns, 2010; Bassett and Sporns, 2017) based on evaluation of pairwise connections between cells or brain areas (Sporns, 2018). Our analyses explore how detection of significant Ca^2+^ events at the level of single cells affects the analyses of network activity based on graph theory methods. To determine the impact of different analysis methodologies on interpretation of experimental outcomes, we utilize a dataset of nucleus accumbens shell imaging results from animals trained to self-administer cocaine. The nucleus accumbens is involved in motivation and drug-seeking behaviors. Multiple studies have shown that cocaine self-administration alters neuronal activity in the nucleus accumbens (Zhang et al., 2002; Pierce and Wolf, 2013; Calipari et al., 2016) and impacts astrocyte morphology (Scofield et al., 2016) with functional and behavioral consequences (Scofield, 2018; Kardos et al., 2019).

## Materials and Methods

### Animal Training

Male Sprague–Dawley rats (*Rattus norvegicus*) weighing 225–250 g were obtained from Taconic Laboratories. Rats were individually housed with food and water available *ad libitum* in their home cages. A 12/12-h light/dark cycle was used with lights on 7:00 am–7:00 pm. All experimental procedures were performed during the light cycle. The experimental protocols were approved by the University of South Carolina and University of Kentucky Animal Care and Use Committees.

Two cohorts of eight rats each were used for the experiments. Both cohorts underwent jugular catheterization surgery: under isoflurane anesthesia (1.5–2.5% isoflurane in O_2_), an indwelling silastic catheter (SAI Infusion technologies, Lake Villa, IL, United States) was placed into the right jugular vein and sutured in place. The catheter was routed subcutaneously to a mesh platform between the shoulder blades. Catheters were flushed daily with 0.3 ml of the antibiotic timentin (0.93 mg/ml) dissolved in heparinized saline. The catheters were sealed with plastic obturators when not in use.

Following catheter implantation, rats were placed in a stereotaxic apparatus under inhalational 1.5–2.5% isoflurane. Genetically encoded calcium indicator (GECI) GCaMP6f targeting either neurons (AAV9.Syn.GCaMP6f.WPRE.SV40, Addgene 100837) or astrocytes (pZac2.1.gfaABC1D.cytoGCamp6f.SV40, Addgene 52925) was injected bilaterally (2 μl/side) into the nucleus accumbens (NAc) shell *via* a Neuros syringe (Hamilton) targeting the following coordinates (relative to bregma): 1.0 mm anterior, ±1.0 mm lateral, and 7.0 mm ventral. Rats began behavioral training 14–20 days after viral microinjections.

In each cohort, four animals underwent cocaine self-administration training and four others served as their yoked saline counterparts. For self-administration training, rats were placed in operant chambers (Med Associates) for 2 h/day for 14–18 days during which time two levers (one “active,” one “inactive”) were extended. For rats undergoing self-administration training, an active lever press resulted in an infusion of cocaine solution (0.21 mg cocaine/50 μl saline over 2.8 s). Each infusion was followed by a 20-s timeout during which lever pressing had no scheduled consequences. All animals started on a fixed ratio (FR) 1 schedule that was increased to FR5 following acquisition of stable responding (<10 variability in active lever presses across three consecutive days). The yoked saline animals received a saline infusion every time their yoke counterpart received cocaine. There were no scheduled consequences to lever pressing by the yoked saline animals. Behavior results are shown in Supplementary Figure 1.

### Ca^2+^ Imaging

After self-administration training, the cohort of animals injected with the neuron-specific GCamp6f (hSyn animals) were sacrificed by guillotine. The brain was excised and 300-μm-thick slices were prepared using a vibratome (Leica VT1200S) in an ice-cold artificial cerebrospinal fluid (aCSF) cutting solution in which NaCl was replaced with an equiosmolar concentration of sucrose. A total of 19 hSyn slices were imaged from seven different rats (3 yoked saline and 4 cocaine) with each rat contributing 1–4 slices to the final dataset. The cohort of animals injected with the astrocyte-targeting GCamp6f (GFAP animals) produced 22 slices from eight different rats with each rat contributing 2–3 slices. The aCSF composition was as follows (in mM): 130 NaCl, 3 KCl, 1.25 NaH_2_PO_4_, 26 NaHCO_3_, 10 glucose, 1 MgCl_2_, and 2 CaCl_2_, pH 7.2–7.4, when saturated with 95% O_2_ and 5% CO_2_; osmolarity was 305–315 mOsm. After cutting, the slices were kept in aCSF heated to 36°C for 30–40 min, following which they were maintained in aCSF at room temperature until transfer to the recording chamber.

Two-minute videos of spontaneous activity in the NAc shell were acquired with an ORCA-Flash 4.0 (V2) digital camera during LED excitation (X-Cite XLED1, Excelitas Technologies). Videos were taken at 512 × 512 pixels and collected with a 40× objective (0.065 μm/pixel) at 25 frames per second.

### Image Analysis

We used a slightly modified version of ABLE (Reynolds et al., 2017) to segment our videos based on activity. The only changes we made were to remove the thresholds (caps) for maximum seed and final region of interest (ROI) size. For our dataset, we used ABLE parameters: alpha = 0.1, lambda = 150 (following Reynolds et al., 2017), and a radius of 20. Modified ABLE scripts are available upon request. The background and neuropil signal were identified for each ROI independently by finding the average (mean) trace in a 60-pixel-wide “halo” around a given ROI, excluding those points found to be in other ROIs. Individual neuropil signals were subtracted from the corresponding ROI traces. Once the ROIs were identified for a recording, those same ROIs and associated fluorescent signal traces were used for all subsequent method comparisons throughout the study. Examples of ABLE-detected ROIs for both neuron and astrocyte populations are presented in Supplementary Figure 2.

### Event Detection

Events were identified from each ROI trace using either a d*F*/*F*_0_ thresholding method or continuous wavelet transform of each trace followed by a custom-written ridgewalking algorithm. To construct d*F*/*F*_0_ traces, we determined *F*_0_ by three different methods: calculating the mean value of an initial window on the trace which we will refer to as “*F*_0_ initial” (e.g., Ellefsen et al., 2014; Lock et al., 2015; Rahmati et al., 2016); finding the smoothed 8th percentile point in a sliding window producing a variable *F*_0_ time series, referred to as “*F*_0_ smooth” (e.g., Patel et al., 2015; Romano et al., 2017; Mancini et al., 2018); and calculating the mean value of a baseline segment found by minimizing the quantity (trace variance + trace mean^2^)^1/2^ in a sliding window on the trace, which we call “*F*_0_ minimal.” We then applied four different thresholding criteria to identify significant activity within a d*F*/*F*_0_ trace: 2.5 standard deviations above the mean of either the baseline (baseline SD) or the total d*F*/*F*_0_ trace (trace SD) and *p*-value of <0.05 as determined by *z*-test of either the baseline (baseline *z*-score) or the total d*F*/*F*_0_ trace (trace *z*-score). The possible combinations of choice of *F*_0_ and thresholding criteria yielded 12 different d*F*/*F*_0_ event detection methods. These methods were compared to a wavelet ridgewalking algorithm that does not require definition of *F*_0_ to identify significant events.

**FIGURE 1:**
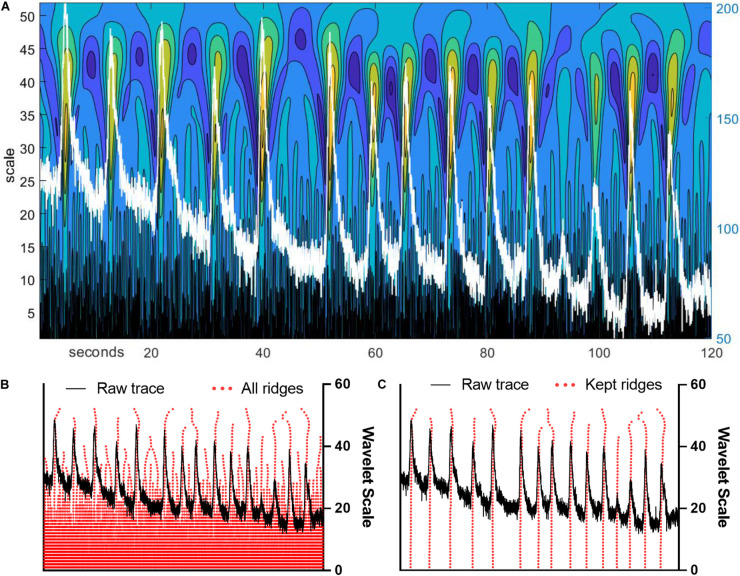
Wavelet ridgewalking algorithm identifies neuronal events with high fidelity. **(A)** Continuous wavelet transform amplitude color map for an example trace overlaid in white. The left *y*-axis is the wavelet scale, the right *y*-axis is the arbitrary units of trace fluorescence amplitude, and the *x*-axis is the recording frame. Color scales are continuous wavelet transform (CWT) values with warmer colors representing larger CWT amplitudes. **(B)** Example trace with ridges generated by ridgewalking algorithm overlaid in red. **(C)** Example trace with significant (kept) ridges overlaid.

Before a wavelet ridgewalking algorithm is used for event detection, a continuous wavelet transform (CWT) is applied to a signal trace producing a two-dimensional sheet of coefficients revealing localized “ridges” of local maxima (Figure 1A). Significant events can be identified by the properties of these ridges (Figures 1B,C), particularly ridge length. The CWT was calculated using the jLab MATLAB toolbox (Lilly, 2019). We used a generalized Morse wavelet with (shape) parameter gamma = 3 and initial (scale) parameter beta = 2 (before scaling). Wavelets having gamma = 3, also called Airy wavelets, were chosen because they have the smallest Heisenberg area (Lilly and Olhede, 2012). We used a set of optimal scales/frequencies determined by jLab scripts for time series of length *N* = 3001, the number of frames in our videos. The completed CWT for a trace results in a two-dimensional sheet of CWT amplitudes, as transform amplitudes for wavelets of all scale sizes are calculated for each time point in the trace. We then generate ridges along the scale dimension using a custom-written MATLAB ridgewalking function. The algorithm is as follows: (a) All local CWT amplitude maxima for *each* scale are identified. These local maxima serve as new ridge initializations and candidates for ridge extension. (b) The local CWT amplitude maxima at the largest scale size are the first ridge initializations. Progressing to smaller scales, local maxima are linked into a ridge if they fall within the temporal range determined by the central window for the wavelet of the preceding scale size when the window is centered on the ridges and ridge initializing maxima at that (larger) scale. If multiple maxima at the smaller scale fall into the range determined for a ridge, only the largest of the new maxima is appended to the ridge. Others within the window initialize new ridges. If a local maximum does not fall into any of the previous ridges’ or maxima’s windows, it also initializes a new ridge. (c) The algorithm ends either after the maxima at the smallest scale have been processed, at which point all local maxima at all scales have been formed into ridges (or identified as single points), or once a user-specified scale has been reached. Gaps in scale along ridges are not allowed. Scale gaps terminate ridges. (d) Once all ridges for a trace are identified, ridge significance is determined by passing through at least 41 scales. Additionally, ridges whose largest CWT maxima occur at or below the 10th scale size are excluded as short noise. Noise parameters were explicitly calculated by noise modeling. MATLAB scripts implementing this algorithm are available upon request.

### Noise Modeling

We generated 10,000 white noise time series using pseudorandom number generators and subjected those time series to the ridgewalking algorithm. We constructed empirical cumulative density functions for ridge length and scale of maximum CWT amplitude along a ridge (event size). We repeated this analysis at several different combinations of noise parameters. From these cumulative density functions, we were able to determine thresholds yielding the desired exclusion rate. For event size, we determined the 98% exclusion threshold to be the 10th scale size. Thus, ridges whose maximum CWT amplitude occurred at a scale less than or equal to 10 were removed as “short noise.” This threshold was found to be stable across parameters, including series length. For ridge length, we chose a threshold length corresponding to 98% exclusion rate (41 scales). These length cutoffs were found to be robust across wide regimes of noise variance and series lengths. Ridges were only kept if they met both the ridge length and max CWT amplitude scale size criteria.

### Rasterization, Calculation of Activity Features, and Network Measures

For d*F*/*F*_0_-processed ROIs, rasters were generated based on one of four different thresholding methods as described above. To create rasters from the wavelet ridgewalking-processed ROI traces, we found the location in time and scale of the maximum CWT amplitude along each significant ridge. Maximum CWT amplitudes were used as event centers. Following rasterization, event frequency was calculated as the number of unique events found in the trace. The amplitude was taken as the maximum d*F*/*F*_0_ value within an event window. For the wavelet ridgewalking algorithm, each event had an associated *F*_0_ value calculated from the segment of the trace immediately preceding the event. Each event trace itself was transformed to d*F*/*F*_0_ using its unique *F*_0_, and the amplitude of an event was defined as the maximum d*F*/*F*_0_ value within the event window. Duration was calculated as the length of a rastered segment. For network analyses, connectivity matrices for the ROIs in each video were generated using the phi coefficient between ROI rasters. The phi coefficient is essentially a correlation coefficient for binarized data series, such as rasters. Phi coefficient values were thresholded at 0.2. Any value below 0.2 was set to zero. Connection matrices were binarized to unweighted and undirected connection matrices. Network measures and distance matrices were calculated using the Brain Connectivity Toolbox (Rubinov and Sporns, 2010). Specifically, we calculated the global clustering coefficient, assortativity, density, modularity, characteristic path length, and network efficiency for identified networks. The global clustering coefficient represents how common clustering is within a network where a “cluster” is formed around a node when many of the nodes connected to it are also connected to each other. Assortativity captures the tendency of network nodes to be connected to other nodes with similar numbers of connections. In an assortative network, nodes with similar numbers of connections preferentially connect to each other, while in a disassortative network, nodes preferentially connect to others with dissimilar numbers of connections. Density is a measure of the number of possible connections that are found to actually exist between nodes; thus, in a very dense network, most nodes may be mutually connected to each other. Modularity captures the tendency of a network to be formed of “modules” or subsets of densely interconnected nodes with more sparse connections between these subsets. Characteristic path length is the typical number of connections required to link one node to another in a network. Network efficiency is the inverse of the characteristic path length, reflecting the efficiency of information transfer throughout a network along the extant connections or paths between nodes.

### Simulated Dataset Generation

Pseudoneuronal and pseudoastrocytic events of randomized duration and frequency were embedded in a noise and data-derived bleaching model. Pseudoneuronal events were either peak values with exponential or linear decays. Peak amplitude was fixed for all events to recreate the mean signal-to-noise ratio (SNR) found in our biological datasets. Durations were varied by changing the decay constant of the exponential or increasing the base of the right triangle. Pseudoastrocytic events were isosceles triangles to simulate astrocytic transients with slower rise times and fixed peak values as for pseudoneuronal events. Event duration was varied by increasing the length of the triangle base. Noise was modeled as random white noise. To model fluorescence bleaching, a linear regression fit to each fluorescence trace was generated. Trace segments determined by the wavelet ridgewalking algorithm to be events were removed from the fit. We then found the mean slope of all fits to all traces which was used to model bleaching present in the simulated datasets. Experimental results also determined the SNR in simulated data. SNR was determined based on events identified by our wavelet ridgewalking algorithm. Each trace background, defined as the portions of the trace not indicated as part of an event, had the bleaching removed by regression. The amplitude of each event was divided by the background noise standard deviation of its trace to determine an SNR for each event. The mean of these SNRs (SNR = 69.9 ± 4.7) was used for modeling. All synthetic time series were of identical length to our recordings (3,001 frames). We varied the frequency of events from 1 to 100 and the duration of events from 1 to 500 frames. All codes are available upon request.

### Statistical Analysis

Analyses were done in Excel 2016 or GraphPad Prism 8. All data are reported as mean ± standard error of the mean and statistical significance threshold was set at *p* < 0.05.

## Results

### Wavelet Analysis Outperforms Other Event Detection Methods

**FIGURE 2:**
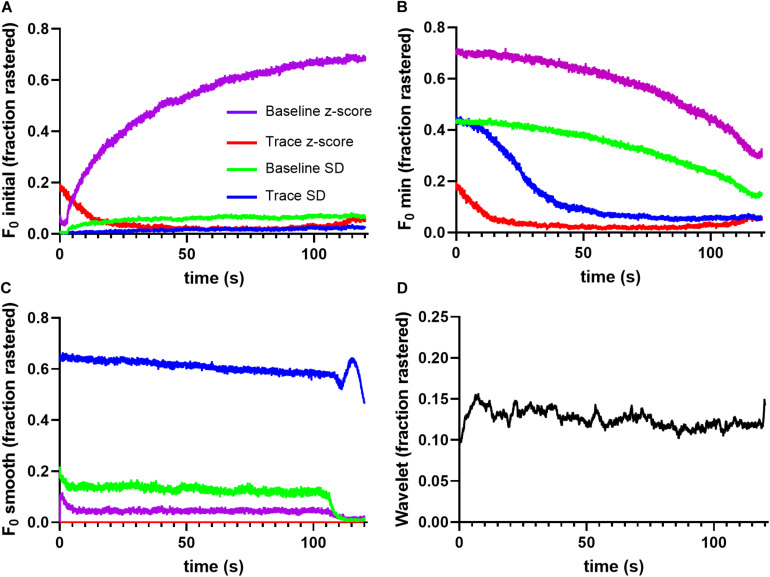
Different event identification methods find different event probabilities. **(A)** F_0_ initial thresholding finds very different, temporally unstable firing probabilities for each thresholding scheme, as does F_0_ minimal in panel **(B)**. **(C)** F_0_ smooth thresholding identifies relatively stable firing probabilities, but the discovered rates vary widely with the thresholding scheme. **(D)** Wavelet ridgewalking algorithm also produces relatively stable firing probabilities.

**FIGURE 3:**
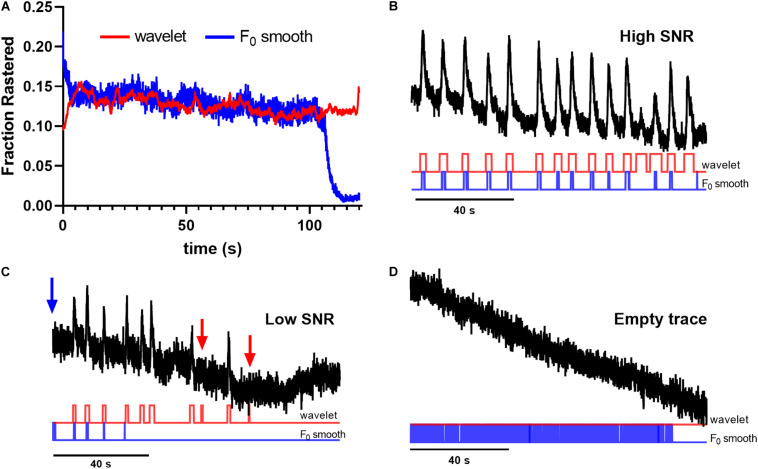
Wavelet analysis outperforms F_0_ smooth thresholding method for event identification. **(A)** Wavelet ridgewalking algorithm and F_0_ smooth thresholding method produce similar raster probabilities. **(B)** In high signal-to-noise ratio (SNR) conditions, the two methods appear to identify almost identical events, but F_0_ smooth finds those events to be shorter than wavelet ridgewalking. **(C)** In lower SNR conditions, the F_0_ smooth thresholding method begins to fail, not identifying short spike events that wavelet ridgewalking readily detects. **(D)** F_0_ smooth fails to identify “dead” ROIs whose traces contain no events, while wavelet analysis can be used to discard them. Red arrows indicate false events captured by wavelet ridgewalking, blue arrow indicates a false event captured by the F_0_ smooth method.

To quantify method performance, we calculated the probability that any ROI displayed significant events in a given image frame using each tested method. We reasoned that for spontaneous neuronal activity, this probability should be stable across time, and that deviations are likely caused by erroneous event identification. Figure 2 illustrates the fraction of ROIs determined to be active at a given time point in the trace. Notice that the *F*_0_ initial and *F*_0_ minimal approaches produced activation probabilities that varied widely across time for the majority of applied thresholds (Figures 2A,B), while the *F*_0_ smooth method produced more stable probabilities (Figure 2C). However, the performance of the *F*_0_ smooth method suffered from a sharply defined degradation toward the end of the trace. Additionally, thresholding by the trace *z*-score failed to identify any significant events in *F*_0_ smooth traces. End-of-time series degradation was likely due to declining number of points in the sliding window required by the *F*_0_ smooth algorithm. The wavelet ridgewalking algorithm also found relatively stable event probabilities (Figure 2D) but did not show the end-of-time series degradation. Overall, the choice of thresholding criterion for the *F*_0_ methods led to as much as a 7-fold difference in the fraction of signals considered to represent significant Ca^2+^ transients (Figures 2A–C). This caveat did not apply to the wavelet ridgewalking method where the event detection criterion has been empirically determined. The *F*_0_ smooth method with a baseline SD threshold and the wavelet ridgewalking method produced event probabilities that converged for a large portion of the time series (Figure 3A). However, we found that while event probabilities were similar, the events captured within the rasters were not the same. For example, under conditions of high SNR (SNR = 27.6892), the wavelet ridgewalking algorithm and *F*_0_ smooth found almost identical events, but the *F*_0_ smooth method found those events to be of shorter duration and fragmented some events. In the example shown in Figure 3B, the *F*_0_ smooth method misses a large event toward the end of the time series, beyond the range of optimal performance for this method. Figure 3C shows an example trace with a lower SNR (SNR = 8.9381) and events of shorter duration. In this example, wavelet analysis outperforms the *F*_0_ smooth method, capturing all visible events, while the *F*_0_ smooth method misses several. Under low SNR conditions, both the wavelet ridgewalking algorithm and *F*_0_ smooth method identified what appeared to be false events which, however, were not the same for the two methods (Figure 3B, arrows). Figure 3D shows an extreme case: a trace that displayed substantial bleaching over the time series and without any recognizable events. For this trace, the wavelet ridgewalking algorithm correctly output an empty raster, while the *F*_0_ smooth method identified events throughout the trace. This result demonstrated a particularly troubling quality for a method to be used in an automated unsupervised pipeline, as such a trace and the ROI associated with it would be kept in the dataset when it should clearly be removed. Therefore, the apparent similarity of performance between the wavelet ridgewalking algorithm and *F*_0_ smooth method is misleading. It is likely that the tendency of the *F*_0_ smooth method to falsely identify events is balancing its propensity for missing true events in such a way as to buoy its rasterization probability (under the baseline SD thresholding criterion) to approximately that of the wavelet ridgewalking algorithm.

### Event Detection Methods Exhibit Differential Performance on Simulated Datasets

**FIGURE 4:**
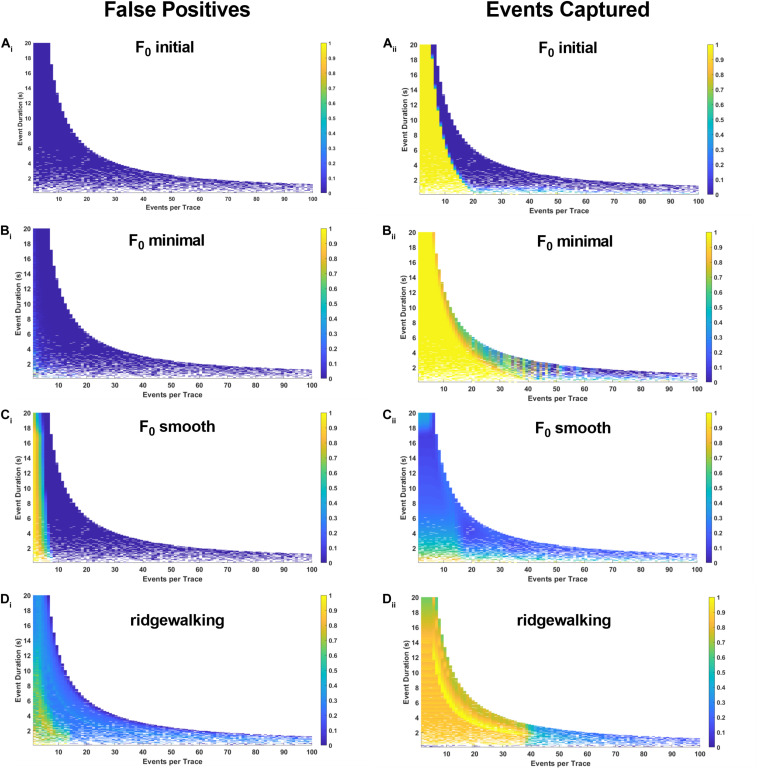
Synthetic data for pseudoneuronal events with linear decay. The left-hand plots represent “false positives” or the fraction of rasters existing outside of known event bounds, and the right-hand plots represent the “events captured” or the fraction of total event bounds that are covered by the rasters. **(A_i,ii_)** Results for F_0_ initial thresholded at 2.5 baseline standard deviations. **(B_i,ii_)** Results for F_0_ minimal thresholded at 2.5 baseline standard deviations. **(C_i,ii_)** Results for F_0_ smooth thresholded at 2.5 baseline standard deviations. **(D_i,ii_)** Results for wavelet ridgewalking algorithm.

**FIGURE 5:**
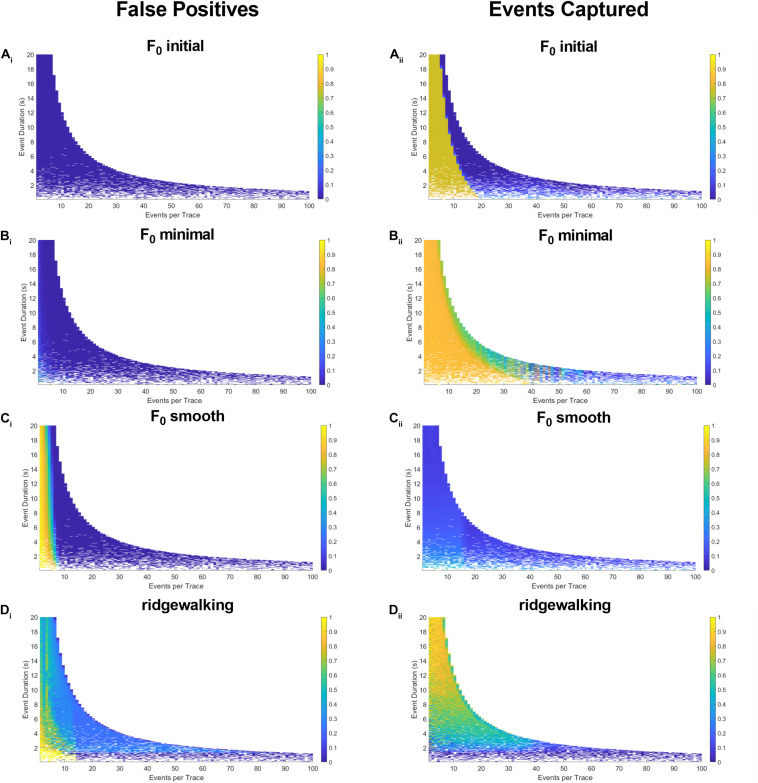
Synthetic data for pseudoneuronal events with exponential decay. The left-hand plots represent “false positives” or the fraction of rasters existing outside of known event bounds, and the right-hand plots represent the “events captured” or the fraction of total event bounds that are covered by the rasters. **(A_i,ii_)** Results for F_0_ initial thresholded at 2.5 baseline standard deviations. **(B_i,ii_)** Results for F_0_ minimal thresholded at 2.5 baseline standard deviations. **(C_i,ii_)** Results for F_0_ smooth thresholded at 2.5 baseline standard deviations. **(D_i,ii_)** Results for wavelet ridgewalking algorithm.

**FIGURE 6:**
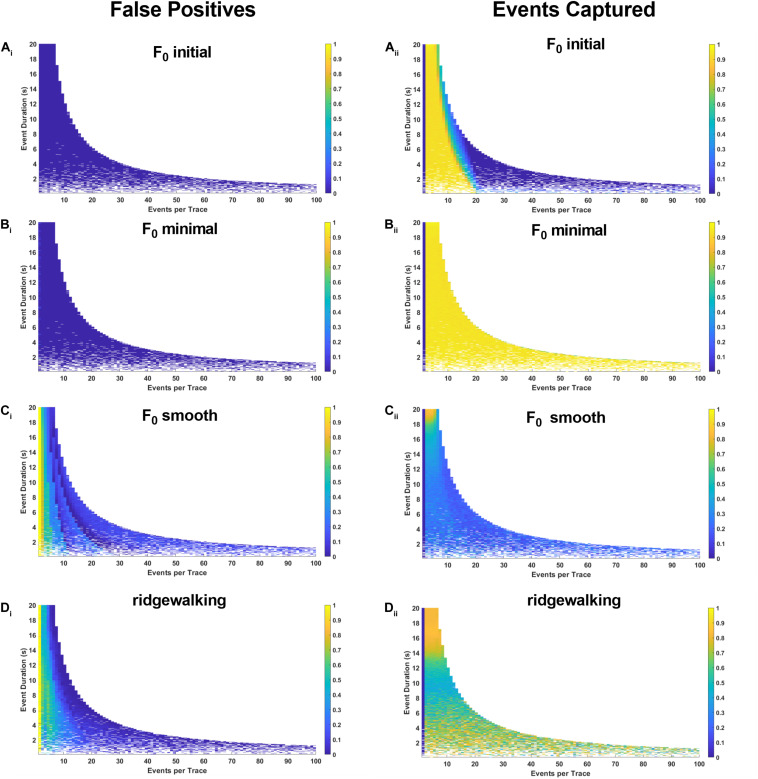
Synthetic data for pseudoastrocytic events. The left-hand plots represent “false positives” or the fraction of rasters existing outside of known event bounds, and the right-hand plots represent the “events captured” or the fraction of total event bounds that are covered by the rasters. **(A_i,ii_)** Results for F_0_ initial thresholded at 2.5 baseline standard deviations. **(B_i,ii_)** Results for F_0_ minimal thresholded at 2.5 baseline standard deviations. **(C_i,ii_)** Results for F_0_ smooth thresholded at 2.5 baseline standard deviations. **(D_i,ii_)** Results for wavelet ridgewalking algorithm.

To more robustly quantify the fidelity with which different methods capture underlying Ca^2+^ transients, we generated model datasets with varying event numbers and widths (see section “Materials and Methods”). Since the number and timing of modeled events were predetermined, we could evaluate the exact rate with which the respective event detection methods were successful in capturing them. The results in Figures 4, 5 for right-triangle (pseudoneuronal) events and isosceles triangle (pseudoastrocytic) events, respectively, show two measures of performance across the four event detection methods: the plots on the left visualize the percent of the rasters that fall outside of the known event bounds (i.e., incorrectly identified events or “false positives”); the plots on the right show the percent of total event durations that are correctly captured by the respective method. The ideal method would exhibit low values on the left panels and high values on the right, indicating a low rate of both false positives as well as a high rate of event capture. In terms of false positives, we found relatively good performance for all methods. *F*_0_ initial and *F*_0_ minimal methods performed ideally (Figures 4A_i_,B_i_), while *F*_0_ smooth and wavelet ridgewalking found some false events in the case of few actual events (Figures 4C_i_,D_i_). In terms of events captured, *F*_0_ initial, *F*_0_ minimal, and wavelet ridgewalking all outperformed *F*_0_ smooth (Figures 4A_ii_–D_ii_). Interestingly, *F*_0_ initial, *F*_0_ minimal, and wavelet approaches were also noted to have well-defined ranges of parameters beyond which their performance sharply declined. For pseudoneuronal events, the *F*_0_ initial method transition line between high and low performance follows a curve from 20 events/trace at the smallest event width to 5 events/trace at the largest event width (Figure 4A_ii_). For *F*_0_ minimal, which has a broader range of high performance, this transition follows a curve from 40 events/trace at the smallest event width to 7 events/trace at the largest (Figure 4B_ii_). Wavelet ridgewalking began to fail when there were more than 40 events in a single trace of 3,001 frames. For right triangular events, wavelet ridgewalking exhibited high performance through the broadest ranges of event frequency and duration (Figure 4D_ii_). We also modeled pseudoneuronal events with exponentially decaying amplitudes (Figure 5). The results followed the same patterns as observed for right triangular events (Figure 4), though all methods captured less of the simulated events (Figures 5A_ii_–D_ii_) and the wavelet ridgewalking algorithm in particular exhibited a marked reduction in performance for events of shorter duration (Figure 5D_*ii*_). We observed generally similar patterns for pseudoastrocytic events modeled as isosceles triangles (Figure 6). *F*_0_ initial and *F*_0_ minimal captured the smallest fraction of false positives (Figures 6A_i_,B_i_) relative to the *F*_0_ smooth and wavelet approaches (Figures 6C_i_,D_i_). The methods varied in the degree to which they successfully captured events (Figures 6A_ii_–D_ii_), and the *F*_0_ minimal method exhibited the highest overall performance for these data, with near-perfect event identification across the majority of the event frequency and duration space probed. These results support marked differences in fidelity of event detection across the examined methods.

### Quantification Method Impacts the Interpretation of Neuronal Ca^2+^ Data

**FIGURE 7:**
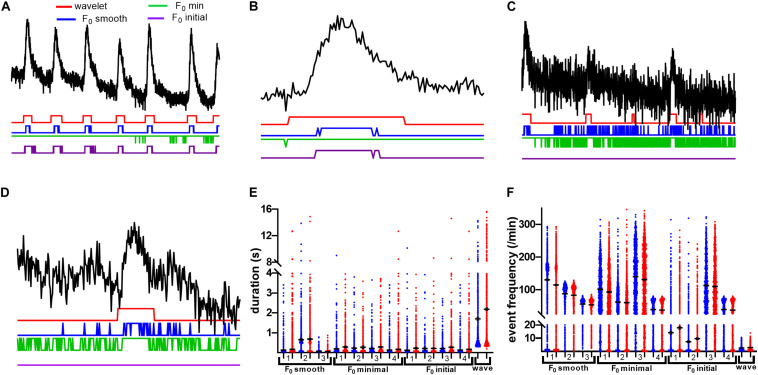
df/F_0_ thresholding methods fragment events with effects on Ca^2+^ activity measures. **(A)** A 1-min sample of neuronal trace, with example rasters beneath. All F_0_ methods were thresholded at 2.5 times the standard deviation of the baseline. Note that, where wavelet finds continuous events, F_0_ methods have a tendency to introduce fragments. Furthermore, in the case of F_0_ minimal, most of this trace is deemed active, with little to no segregation between events. **(B)** A zoomed short segment of a neuronal trace with high SNR. Notice that even in high SNR conditions, fragmentation still occurs. **(C)** A 1-min sample of a low SNR neuronal trace with example rasters beneath. All F_0_ methods were thresholded as in **(A)**. With low SNR, there is increased fragmentation by F_0_ smooth and F_0_ minimal methods. F_0_ initial thresholding fails utterly for this trace. **(D)** A zoomed short segment of a neuronal trace with low SNR. F_0_ initial fails to identify this event. **(E)** Distributions of mean event durations for presumed neurons from saline and cocaine self-administration groups as calculated by the various methods. The number for each method indicates thresholding criteria as follows: 1, baseline sd*2.5; 2, trace sd*2.5; 3, *z*-score baseline *p* = 0.05; 4, trace *z*-score *p* = 0.05. Note that the wavelet method finds events to be of longer duration due to lack of fragmentation effects. **(F)** Neuronal event frequency distributions. All F_0_ thresholding methods identify several times more events than the wavelet ridgewalking method for the same traces.

We noticed a general susceptibility of the d*F*/*F*_0_-based event detection methods to two types of errors: event fragmentation and introduction of spurious events at time points that differed between methods (Figures 7A–D). It is tempting to ascribe the short fragments to noise and discard them; however, we found that many of these fragments occur within visible event boundaries (Figures 7B,D), indicating that *F*_0_-based approaches would generally benefit from postprocessing to determine which events to exclude and which to merge together for a faithful reflection of neuronal activity. Additionally, the *F*_0_ initial and *F*_0_ minimum baseline methods appeared to introduce large-scale time structure to rasters produced from our dataset (cf. Figures 2A,B). Where we expected relatively stable event probability for spontaneous transients, these methods exhibit pronounced changes in event probability as time progresses. These large-scale effects may be due to the selected methods’ inabilities to account for fluorescence bleaching present during recording. The variability between methods that we observe could introduce substantial uncertainty into the interpretation of data related to frequency and duration of Ca^2+^ transients. Figures 7E,F show the distributions of event durations and event frequencies found by each of the tested methods. Most methods found substantially different mean frequencies and distributions. Even when the same definition of *F*_0_ is used, the choice of threshold alters the distribution mean and shape. We note that across the board, d*F*/*F*_0_ thresholding methods found shorter mean event durations and higher mean event frequencies, both of which can be traced back to those methods’ tendencies to fragment events and misidentify spurious segments as significant transients.

To further evaluate the extent to which the choice of event detection algorithm impacts the interpretation of experimental data, we compared the characteristic features of neuronal Ca^2+^ transients: amplitude, frequency, and duration of events. The data were split into imaging sessions from animals trained to self-administer cocaine and their yoked saline controls. Characteristic features were examined at three population scales: individual events across all ROIs (saline: *N*_*events*_ = 5,454–287,100; cocaine: *N*_*events*_ = 9,353–408,411), mean values for each ROI across all slices (saline *N*_*ROI*_ = 1,025; cocaine N_*ROI*_ = 1,556), and mean values for each slice across all animals (saline *N*_*slice*_ = 7; cocaine *N*_*slice*_ = 12). The summary of our findings is represented in Table 1. Significant differences between saline and cocaine populations were determined by Mann–Whitney *U* tests. Most methods found significant differences in event duration between saline and cocaine self-administration conditions at the level of pooled events. The significant differences found between conditions declined at the next tier of analysis (pooled ROI means) and vanished altogether at the level of pooled slice means. Considering that the direction of changes among statistically significant findings remained the same, this effect was likely driven by diminishing sample size at progressively higher levels of analysis. Encouragingly, all methods with the exception of *F*_0_ smooth under 2.5 times total trace standard deviation threshold found the same qualitative effect of cocaine self-administration: increased event duration.

**TABLE 1:**
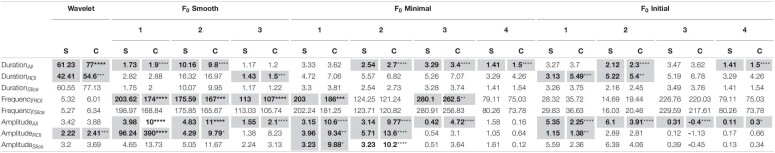
Mean values of neuronal calcium event measures found using each of the tested methods.

*p < 0.05; **p < 0.01; ***p < 0.001; ****p < 0.0001. Significant differences were determined by Mann–Whitney U test. Shaded cells with bolded values indicate significantly different saline/cocaine group pairs.

Some of the d*F*/*F*_0_ thresholding methods identified significant decreases in event frequency associated with cocaine self-administration at the level of the ROI means (Table 1). In general, the d*F*/*F*_0_ methods found dramatically more events than the wavelet method at both the cell population and slice mean levels of analysis, which, combined with the event duration results, supports our finding that d*F*/*F*_0_ thresholding methods are capturing multiple short fragments of events compared with wavelet analysis’ tendency to capture and represent events in a single segment.

Relative fluorescence amplitude is one of the most widely reported measures of Ca^2+^ activity. We found that most methods with the exception of the wavelet ridgewalking algorithm reported significant differences in event amplitude in the cocaine self-administration group at the level of individual events. However, the different methods disagreed on whether cocaine self-administration leads to increased or decreased event amplitude (Table 1). The results became more homogeneous at the level of the ROI means, with cocaine self-administration being found to produce higher-amplitude events, but the quantitative value of this difference varied substantially. For example, some negative mean amplitude values were reported for the *F*_0_ initial method with a baseline *z*-score threshold, which could be caused by failure to appropriately account for decreasing fluorescence due to bleaching. Additionally, the scale of analysis for the *F*_0_ smooth method under a 2.5 baseline standard deviation method impacted the calculated amplitudes dramatically with the mean amplitude of all ROIs being much higher than the slice mean or events mean. This reflects a tendency to identify a subset of ROIs to have sparse but very high amplitude events compared to the general findings of this method. With few exceptions, the effects of cocaine self-administration appeared magnified when analyzed by the *F*_0_ methods relative to the wavelet analysis at every scale.

We performed Kruskal–Wallis tests to determine which methods produced the most disparate results. These analyses were done only at the level of ROI means. For event durations, we found that out of 66 pairwise comparisons, 59 differed significantly from each other in both saline and cocaine self-administration conditions, and those methods that were not statistically different from each other were identical for the saline and cocaine datasets (Supplementary Table 1). Similar to event durations, event frequency data varied significantly across most methods. Out of eight methods that produced similar results in the saline group and seven methods that produced similar results in the cocaine group, five were shared between groups (Supplementary Table 2). The differences in amplitude of detected events were also significant across most of the methods and 12 comparisons that did not reach significance were shared between the saline and cocaine groups (Supplementary Table 3).

### Quantification Method Impacts the Interpretation of Astrocytic Ca^2+^ Data

**FIGURE 8:**
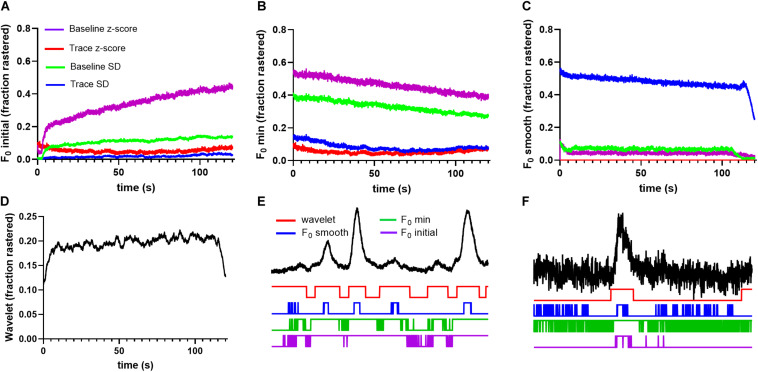
Event detection methods find highly variable raster probabilities for astrocytic recordings. **(A)** F_0_ initial thresholding methods produce largely unstable results with varying raster probabilities, as do F_0_ minimal thresholding methods in **(B)**. **(C)** F_0_ smooth produces stable probabilities, but those probabilities vary between thresholds. **(D)** Wavelet ridgewalking identifies stable raster probability that is slightly higher than found for neurons. Long duration of astrocytic events makes them more likely to be cut off at the beginning and end of time series leading to a drop in probability of detection by wavelet ridgewalking. **(E)** A 1-min trace of high SNR astrocyte fluorescence trace with example rasters beneath. Even in these high SNR conditions, the F_0_ thresholding methods introduce fragments. The F_0_ smooth method, which performed well identifying calcium transients in high SNR neuronal traces, does worse with astrocytic traces as seen in this example, probably due to their relatively slower kinetics. **(F)** A 1-min segment of a low SNR astrocytic trace taken from above the calculated rasters. Once again, in low SNR conditions, the F_0_ thresholding methods introduce multiple fragments.

Our synthetic data (Figures 4–6) indicated that differences in kinetics of Ca^2+^ events may have an impact on the relative performance of event detection algorithms. Therefore, we compared the method performance when applied to the analysis of astrocytic Ca^2+^ transients which are substantially more heterogeneous than neuronal events and exhibit generally slower rise and decay times. As for the neuronal recordings, we began by analyzing the probability of astrocytic ROI activation found by each method based on events pooled across saline and cocaine conditions. The probability to detect an event varied as a function of time for *F*_0_ initial and *F*_0_ minimal approaches, although the magnitude of variation was less than exhibited for neurons (Figures 8A,B). Once again, the *F*_0_ smooth and wavelet ridgewalking algorithm displayed the most stable performance across time. However, in the case of astrocytes, activation probabilities were dissimilar between these two methods, with the wavelet ridgewalking algorithm finding a slightly increased probability of activation for astrocytes relative to the *F*_0_ smooth algorithm (Figures 8C,D). Event fragmentation and foreshortening of event duration caused by raster fragments were also obvious with the astrocyte data (Figures 8E,F).

To examine how these discrepancies would impact the interpretation of experimental manipulations, we compared frequency, amplitude, and duration of astrocytic Ca^2+^ transients in brain slices of animals trained to self-administer cocaine and saline controls. The wavelet ridgewalking method identified astrocytic Ca^2+^ transients as tending to be significantly longer than those observed in neuronal recordings. The d*F*/*F*_0_ methods found mean astrocytic calcium transients to have durations that are of similar, larger, or smaller magnitude than those found for neuronal transients, depending on the method (Table 2). All methods identified significant differences in event duration between the saline and cocaine self-administration populations when all events from each condition were pooled, and most methods converged on the same qualitative conclusion: cocaine self-administration leads to shorter calcium transients in astrocytes. The single exception was the *F*_0_ smooth method used with a trace SD threshold which found that cocaine exposure was associated with an increase in duration of astrocytic transients. All significant differences for event duration between the saline and cocaine groups vanished across all methods when the populations of ROI mean durations or slice mean durations were compared.

**TABLE 2:**
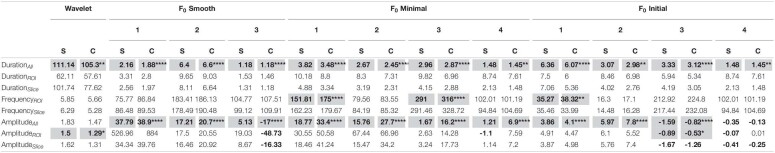
Mean values of astrocytic calcium event measures found using each of the tested methods.

*p < 0.05; **p < 0.01; ***p < 0.001. Significant differences were determined by Mann–Whitney U test. Shaded cells with bolded values indicate significantly different saline/cocaine group pairs.

Three d*F*/*F*_0_ thresholding methods identified differences in frequency between the total population of ROIs converging on an increase in event frequency following cocaine self-administration (Table 2). The other nine methods failed to detect any significant frequency differences. With regard to event amplitude, most d*F*/*F*_0_ thresholding methods found significant differences between saline and cocaine self-administration condition at the level of all events with some of the identified differences contradicting qualitatively (Table 2). Only two methods found significant differences in amplitude at the level of ROI mean values: wavelet ridgewalking, which reported a significant decrease in event amplitude in the cocaine group, and *F*_0_ initial thresholded by baseline *z*-score, which reported a significant increase in amplitude.

### Implications for the Interpretation of Network Structure

One of the advantages of Ca^2+^ imaging is that it allows researchers to probe relationships between multiple cells comprising a local network. We analyzed the impact of event detection methods on neuronal network structure by calculating the global clustering coefficient, assortativity, density, modularity, characteristic path length, and network efficiency for networks found in our slice recordings. Table 3 contains the summary results for neuronal networks. While the majority of methods found no significant differences for any of these measures, the mean values varied widely across methods. For example, network modularity, a measure of connection density within versus outside of detected modules, was qualitatively different between *F*_0_ minimal (baseline SD) and *F*_0_ initial (baseline SD) with the former detecting a significant increase and the latter reporting a decrease in modularity associated with cocaine self-administration.

**TABLE 3:**
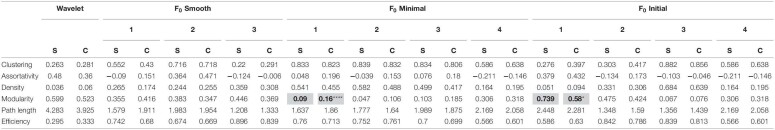
Neuron network measures found using each of the tested methods.

*p < 0.05; ****p < 0.0001. Significant differences were determined by Mann–Whitney U test. Shaded cells with bolded values indicate significantly different saline/cocaine group pairs.

We also carried out the analysis of network measures for astrocytic networks. The results are summarized in Table 4. As with neurons, the mean values of the network measures varied by method, and most methods did not find any significant differences between the two populations. Looking at the characteristic path length and global efficiency, network measures with the strongest trends toward significance, we found that the directionality of the relationship between the two populations is opposite for the wavelet ridgewalking algorithm and d*F*/*F*_0_ thresholding methods: wavelet ridgewalking reported increased characteristic path length and thus decreased global network efficiency associated with cocaine self-administration, while all the *F*_0_ minimal methods and the *F*_0_ initial (*z*-test thresholds) methods found the opposite.

**TABLE 4:**
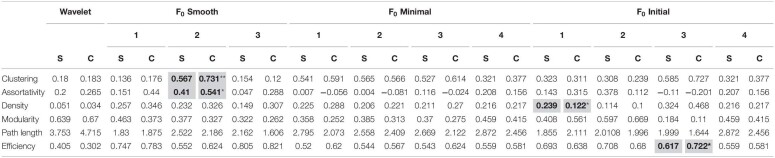
Astrocyte network measures found using each of the tested methods.

*p < 0.05; **p < 0.01. Significant differences were determined by Mann–Whitney U test. Shaded cells with bolded values indicate significantly different saline/cocaine group pairs.

Comparing across methods, we found significant differences between d*F*/*F*_0_ methods and wavelet ridgewalking as well as among the d*F*/*F*_0_ methods themselves for characteristic path length. In the cocaine condition, all significant differences were found to be between the wavelet ridgewalking algorithm and d*F*/*F*_0_ thresholding methods. For network efficiency, there were very few significant differences found in the saline condition, but the wavelet ridgewalking results for the cocaine self-administration population were found to be significantly different from all but one of the d*F*/*F*_0_ methods analyzed (data not shown). Thus, we found that predominant differences for characteristic path length and global network efficiency were between the wavelet ridgewalking method and the d*F*/*F*_0_ thresholding methods, which was the case for most of the network measures analyzed. For both neurons and astrocytes, the differences between wavelet ridgewalking and d*F*/*F*_0_ thresholding method network measures were exacerbated in the cocaine self-administration population (data not shown).

## Discussion

### Imaging Method

Widefield fluorescence microscopy allows the simultaneous visualization of fluorescence from multiple sources across a large area. Superior temporal resolution of widefield microscopy has benefits for the interpretation of relationships between dynamic events, such as intracellular Ca^2+^ transients. However, widefield imaging is vulnerable to contamination by signals from neighboring areas within the field of view as well as by signals originating both below and above the optical imaging plane. While the development of robust automatic image segmentation was not the goal of this study, we attempted to minimize signal contamination by using a modified version of the ABLE algorithm (Reynolds et al., 2017). In ABLE, the initial “seed” ROIs are evolved through algorithmic iterations based on correlated activity between neighboring pixels to produce final ROIs each of which contains all the pixels whose fluorescence activity was significantly similar. Fluorescence from a single pixel is allowed to contribute to any number of ROIs such that, ultimately, uncorrelated signal is excluded while distinct cells remain detectable even in the presence of structural overlap. However, it must also be borne in mind that when discrete domains are capable of generating isolated signals within the soma of a single cell, as is the case for astrocytic Ca^2+^ (Semyanov et al., 2020), the ability of the ABLE algorithm to identify signal overlaps is offset by failure to predict which of those overlaps belong to discrete cells and whether fluorescence spreading through the cell soma is identified as a single or multiple ROIs. This caveat, however, was not explicitly tested in our study. In the context of an automated analysis pipeline, this activity-based approach lends some confidence that only active pixels are included within the ROI outlines and is distinct from approaches that identify ROIs based on thresholds or watershed transforms applied to an average or maximal imaged field (Wong et al., 2010; Shen et al., 2018) or based on secondary fluorescent markers (Wardill et al., 2013). To further reduce potential signal contamination, we removed the local, rather than global, neuropil signal from each individual ROI as has been done in previous studies (Keemink et al., 2018; Pnevmatikakis, 2019; Soltanian-Zadeh et al., 2019). We did not seek to remove the commonly observed bleaching effects from the recordings, although various methods for doing so have been implemented by other groups (Balkenius et al., 2015; Shkryl, 2020). Our results indicate that wavelet ridgewalking outperformed all tested d*F*/*F*_0_ thresholding methods in terms of faithfully identifying events within our *ex vivo* recording data, even in the presence of bleaching.

We observed considerable variability in SNR in our recordings, both within and between the imaged fields. The SNRs of our data were in many cases below SNRs achieved with single-photon or multiphoton confocal systems (Weisenburger et al., 2017; Song, 2019). Our results highlight the sensitivity of the various event detection methods to variability in SNR (e.g., Figures 3, 7). In general, we found that the performance of the wavelet ridgewalking algorithm was on par with or better than the d*F*/*F*_0_ approaches at high SNRs. At lower SNRs, the d*F*/*F*_0_ thresholding methods tended to identify an increasing number of spurious events, while the performance of the wavelet ridgewalking algorithm remained stable.

### dF/F_0_ Thresholding

Our results indicate that the definition of *F*_0_ as well as the choice of event detection threshold has major impacts on d*F*/*F*_0_-based analyses. For example, event frequency differed by as much as 50-fold across detection methods and by as much as 15-fold between different choices of threshold applied with identical definitions of *F*_0_ (Figure 7 and Table 1). Similarly, a broad range of values were found across the analyses of event duration and amplitude. Amplitude-based approaches such as *F*_0_ must take special care to ensure faithful detection of fluorescence fluctuations in part because all other measures are linked to amplitude by definition. In our analyses, we applied the tested methods to traces with unstable fluorescence baselines common in widefield imaging, and our recordings were taken under spontaneous firing conditions. It is possible that further pre- or postprocessing may cause the event identification methods tested to converge on a common set of events and thus produce more similar activity feature results. We did not undertake any preprocessing beyond local neuropil removal and undertook no method-specific postprocessing.

In studies that monitor Ca^2+^ activity triggered by external stimuli (e.g., electrical stimulation, drug application, and behavioral events), normalization of a recorded trace to the fluorescence signal immediately prior to stimulation (e.g., Ellefsen et al., 2014; Lock et al., 2015; Rahmati et al., 2016) presents a reasonable solution for amplitude-based detection, but this cannot be applied to spontaneous fluorescence since imaging starts at an undetermined point with regard to cell activity profile. Moreover, quantification of frequency and duration is less likely to benefit from stimulus-based approaches because they do not solve the problem of event fragmentation that our results indicate are common to *F*_0_-based approaches.

In the presence of fluorophore bleaching, identifying a single section of trace to serve as a baseline *F*_0_ is particularly problematic. Among the event detection methods tested, we found that *F*_0_ minimal and *F*_0_ initial methods had the greatest tendency to introduce large fluctuations in the probability of identifying an event at a given time throughout a trace. These results are unsurprising since both methods assume that fluorescence in a fixed segment of time represents the baseline activity level of the entire trace. Theoretically, *F*_0_ minimal should be superior to *F*_0_ initial as it aims to minimize the likelihood of categorizing active sections of the trace and high-frequency noise as baseline. This was confirmed by simulated data (Figures 4–6). However, in practice, we found that *F*_0_ minimal also tended to overidentify events and did not adequately account for bleaching or low-frequency oscillations (e.g., Figure 7). Wavelet ridgewalking analysis avoids these problems by identifying events independent of trace baselines. *F*_0_ can thus be determined for each transient individually subsequent to event identification. The section of trace associated with each particular transient can then be transformed into d*F*/*F*_0_ to provide a relative amplitude as we have done for our dataset.

In the context of an automated algorithm, there is in general no reason that an event amplitude threshold should be the sole determinant of significant events. The disparity in the performance of different detection methods on biological and simulated traces exemplify this issue. There is a clear distinction between high and low performance for these methods across the spectrum of event widths and frequencies in our simulated data. For example, *F*_0_ initial and *F*_0_ minimal both performed well with simulated data. Within their respective high-performance domains, both methods captured nearly all of the pseudoneuronal events successfully with minimal introduction of falsely identified events. However, despite the encouraging results with the simulated datasets (within their high-performance regimes), both the *F*_0_ initial and *F*_0_ minimal performed poorly with traces extracted from biological data. We believe this to be due to a combination of signal-to-noise variability in biological but not simulated data as well as the nature of “noise” in the two types of data. Thresholds that rely on standard deviation from the mean are predicted to perform well when the noise has a regular/stationary distribution such as white or Gaussian distributed noise. When the background noise is non-stationary and cannot be approximated by such a distribution, the methods may fail for all traces, as shown in our results based on biological data (e.g., Figures 2, 7, 8).

### Wavelet Ridgewalking

Wavelet ridgewalking avoids both the problem of defining *F*_0_ and the problem of threshold selection. In addition, we found that it was resilient to fluctuations in signal-to-noise ratio and bleaching effects. However, our results suggest that the performance of wavelet ridgewalking algorithms may be limited by the frequency of Ca^2+^ transients. For example, in our simulation of pseudoneuronal events, performance of the wavelet ridgewalking algorithm degraded abruptly when there were more than 40 events (of any duration) in the modeled time series (Figure 4D_ii_). We have not explored the extent to which the problem persists across a range of time series lengths, as all simulated time series were constructed to be the same length as our recordings (3,001 frames). The possibility that wavelet ridgewalking algorithm-based event detection methods may fail when trying to image cells with very high frequencies of calcium transients should be kept in mind.

Technically, the wavelet ridgewalking algorithm employs thresholds as well. In our analyses, we removed ridges below a 98% confidence threshold as determined by white noise modeling. However, this determination is not simply based on amplitude but rather represents a 98% confidence that a particular transient is not the product of white noise fluctuations regardless of transient shape, amplitude, or duration. Better or more thorough noise modeling may improve the performance of wavelet analysis further, as it is possible that noise artifacts are identified as significant events because they do not conform to the white noise assumptions.

### Other Event Identification Methods

There are, of course, many other event detection tools available for Ca^2+^ imaging data, some of them also incorporating automatic ROI detection (e.g., CALIMA: Radstake et al., 2019; CaImAn: Giovannucci et al., 2019). Our intent was not to provide an exhaustive comparison between all available tools, but to highlight limitations associated with the use of classic d*F*/*F*_0_ approaches, particularly relative to the wavelet ridgewalking algorithm as an alternative. We avoided library look-up methods (e.g., FluoroSNNAP, Patel et al., 2015) in our comparisons because they require foreknowledge of event shape, which may limit method versatility when applied to morphologically heterogeneous Ca^2+^ transients such as those displayed by astrocytes. Other nuanced methods, such as CNMF (constrained non-negative matrix factorization), are gaining popularity. CNMF (Pnevmatikakis et al., 2016) identifies ROIs, demixes overlapping ROI components, and denoises and deconvolves neuronal spiking activity from fluorescent traces in one fell swoop. CNMF is optimized for two-photon or light sheet microscopy and often has poor performance when used with more complicated background signals (Barbera et al., 2016), such as present in our recordings. CNMF-E is designed for microendoscopic data, which displays a more complicated profile of background signals (Zhou et al., 2018). Both CNMF and CNMF-E are optimized for the detection of neuronal signals, and it is unclear how well these methods could be adapted for analysis of astrocytic calcium transients. Nevertheless, CNMF-E should be appropriate for the analysis of widefield imaging data, and it may be interesting to evaluate its performance relative to wavelet ridgewalking in the future. Even in the absence of testing the breadth of extant event detection methods, our findings indicate that approaches relying on d*F*/*F*_0_ thresholding must be subject to rigorous testing to justify the choice of baseline and event threshold.

### Consequences for the Interpretation of Experimental Data

Our data indicate that the choice of an event detection algorithm has a substantial impact on the interpretation of experimental data both quantitatively and qualitatively. Quantitatively, the measured values of amplitude, frequency, and duration calculated from the same set of ROIs and traces varied significantly between methods even when similar baselines or detection thresholds were used (Supplementary Tables 1–6). These differences could lead to conflicting or even opposite conclusions. For example, in our dataset, cocaine self-administration was found to either suppress or increase the amplitude of neuronal Ca^2+^ transients depending on which approach was chosen to analyze the data. Examples of values trending in opposite direction between saline and cocaine groups could also be found in the analyses of event duration and frequency, although among significant findings, all methods agreed that event duration increased and event frequency decreased in neurons following cocaine self-administration. The convergent findings of decreased frequency are consistent with the reports that action potential firing is reduced after cocaine self-administration (Peoples et al., 2007; Mu et al., 2010). Combined with an increased Ca^2+^ event amplitude, this finding could be interpreted to indicate an elevated number of action potential bursts in the NAc MSNs on the background of general neuronal hypoactivity. This interpretation would have to be modified if cocaine-induced decrease in amplitude was concluded based, for example, on the data from several *F*_0_ initial methods (Table 1).

The results from imaging of astrocytes support our main conclusion: The effect of any experimental manipulation on Ca^2+^ fluorescence can only be ascertained with confidence if the analytical method used faithfully interprets the underlying signal. Automated analysis of astrocytic Ca^2+^ transients is further complicated not only by substantial variability in event shape but also by ambiguities with respect to spatial distribution within the soma of a single cell. Astrocytic Ca^2+^ transients are commonly observed in multiple discrete or overlapping microdomains within a single cell (Khakh and McCarthy, 2015; Agarwal et al., 2017; Verkhratsky and Nedergaard, 2018). Combined with the possibility that astrocytic Ca^2+^ may propagate across cellular subdomains, this poses significant analytic challenges especially since the mechanisms regulating spatial constraints on microdomain signals remain unknown. These considerations additionally pose the problem of image segmentation in stark relief. Activity-based methods of ROI identification, such as the one utilized in our study, may be effective in isolating microdomain signals but might not attribute them to single cells. The problem is amplified by extensive branching of astrocytic processes which may result in small “specks” of activity dispersed throughout the imaged field, a particular challenge in widefield microscopy experiments.

The ability to evaluate activity across multiple cells simultaneously afforded by Ca^2+^ imaging presents an opportunity to examine interactions within cellular networks. We analyzed functional connectivity between the ROIs in our recordings, meaning that we identified connections between ROIs whose rasters were significantly correlated in time. This generates networks of ROIs that are linked by similarities in their activity patterns. However, our approach does not establish effective (causal) or structural (physical) connections between the imaged cells though these are also important for network function. These could be calculated using a lagged phi coefficient, mutual information, or other measures of connectivity and further enhanced by differential labeling and multichannel fluorescence recording (Garofalo et al., 2009; Zhou et al., 2009; Rubinov and Sporns, 2010; Timme and Lapish, 2018). We acknowledge that the power of our slice network analyses may be limited by a relatively small number of slices represented in the saline and cocaine datasets. Ultimately, the disparities in network analysis results that we found here could be traced to the disparities in Ca^2+^ transient identification between the methods used, as all networks were formed based on pairwise comparison of the rasters *via* the phi coefficient. Beyond discrepancies in the identification of specific events, the introduction of any non-random structure to rasters by a particular method such as exhibited in Figures 2, 8 will artificially increase correlation values between rasters, leading to differences in network measures. Effective Ca^2+^ transient identification is, therefore, the crux not only of fluorescent signal activity analyses but for any functional network analysis as well.

## Data Availability Statement

The raw data supporting the conclusions of this article will be made available by the authors, without undue reservation.

## Ethics Statement

The animal study was reviewed and approved by the University of South Carolina and University of Kentucky Animal Care and Use Committees.

## Author Contributions

AN and PO designed the study. AN performed the data analysis, wrote all the codes, and wrote the manuscript. BO’D collected the data used in the analyses. PO wrote and edited the manuscript. All authors contributed to the article and approved the submitted version.

## Conflict of Interest

The authors declare that the research was conducted in the absence of any commercial or financial relationships that could be construed as a potential conflict of interest.
