# Supplementary material for: Comparative Effects of Event Detection Methods on the Analysis and Interpretation of Ca2+ Imaging Data
Source: Front Neurosci. 2021 Mar 26;15:620869. doi: 10.3389/fnins.2021.620869 (PMC8032960; doi:10.3389/fnins.2021.620869)
Supplement: Supplementary file 1 [file Presentation_1.pptx]

## Slide 1
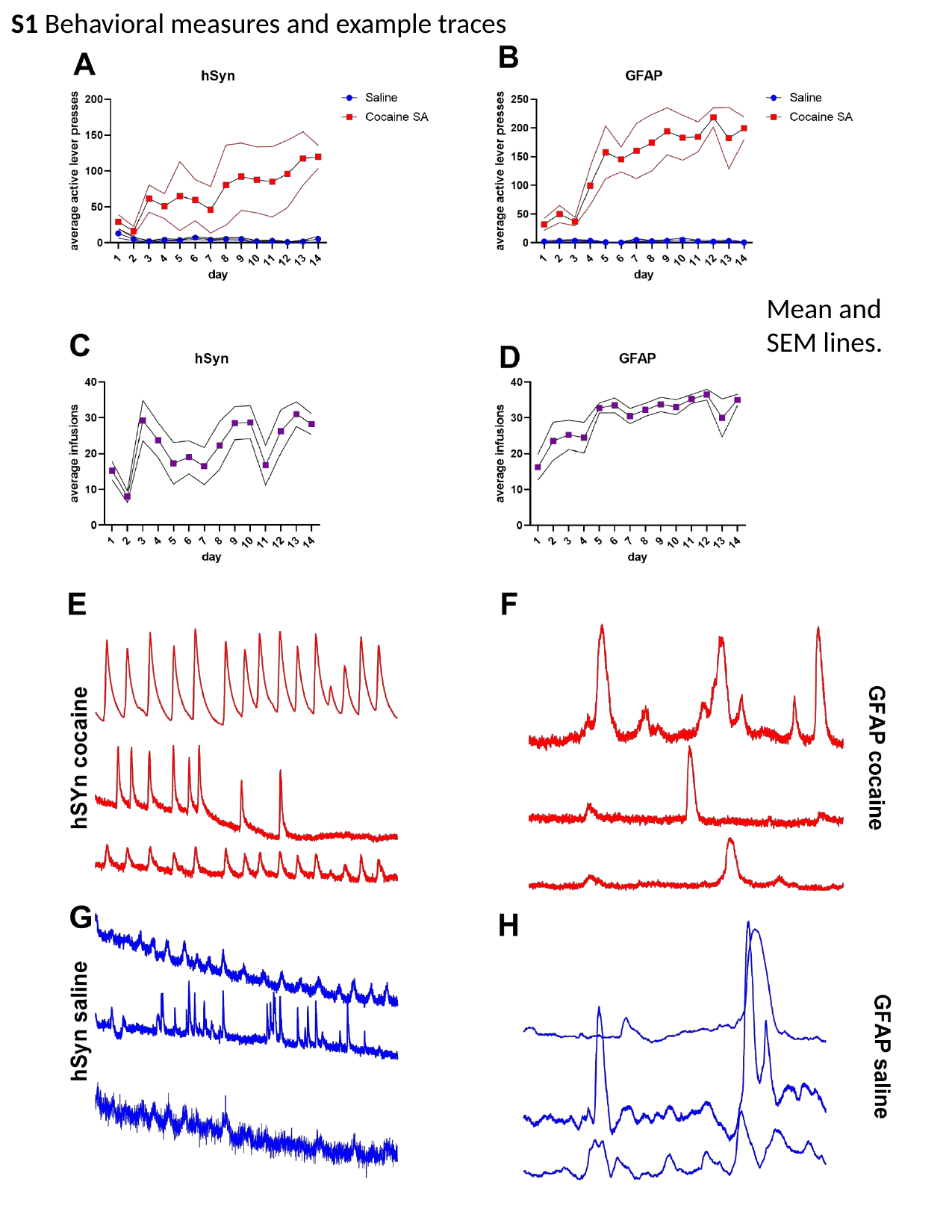

S1 Behavioral measures and example traces
Mean and SEM lines.

## Slide 2
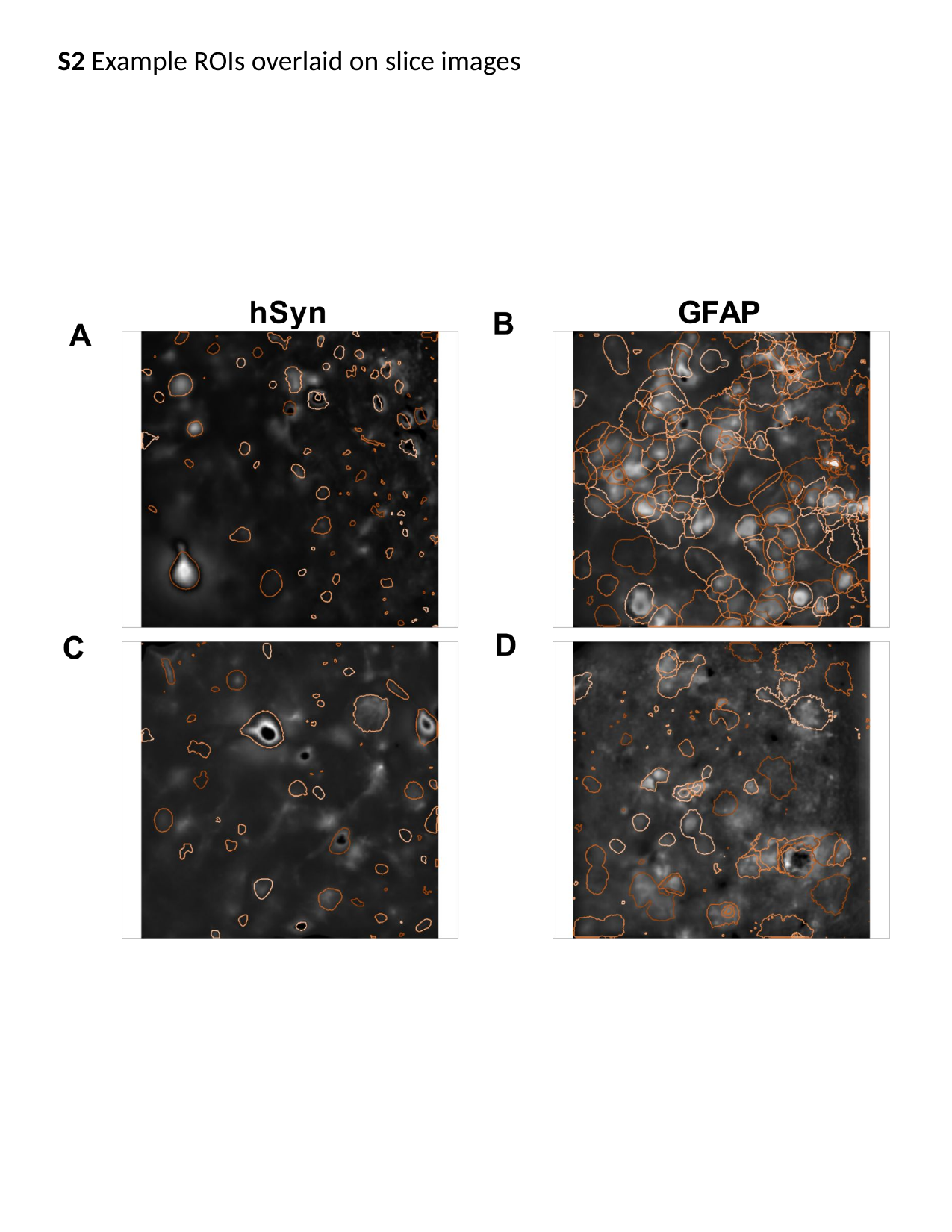

S2 Example ROIs overlaid on slice images

## Slide 3
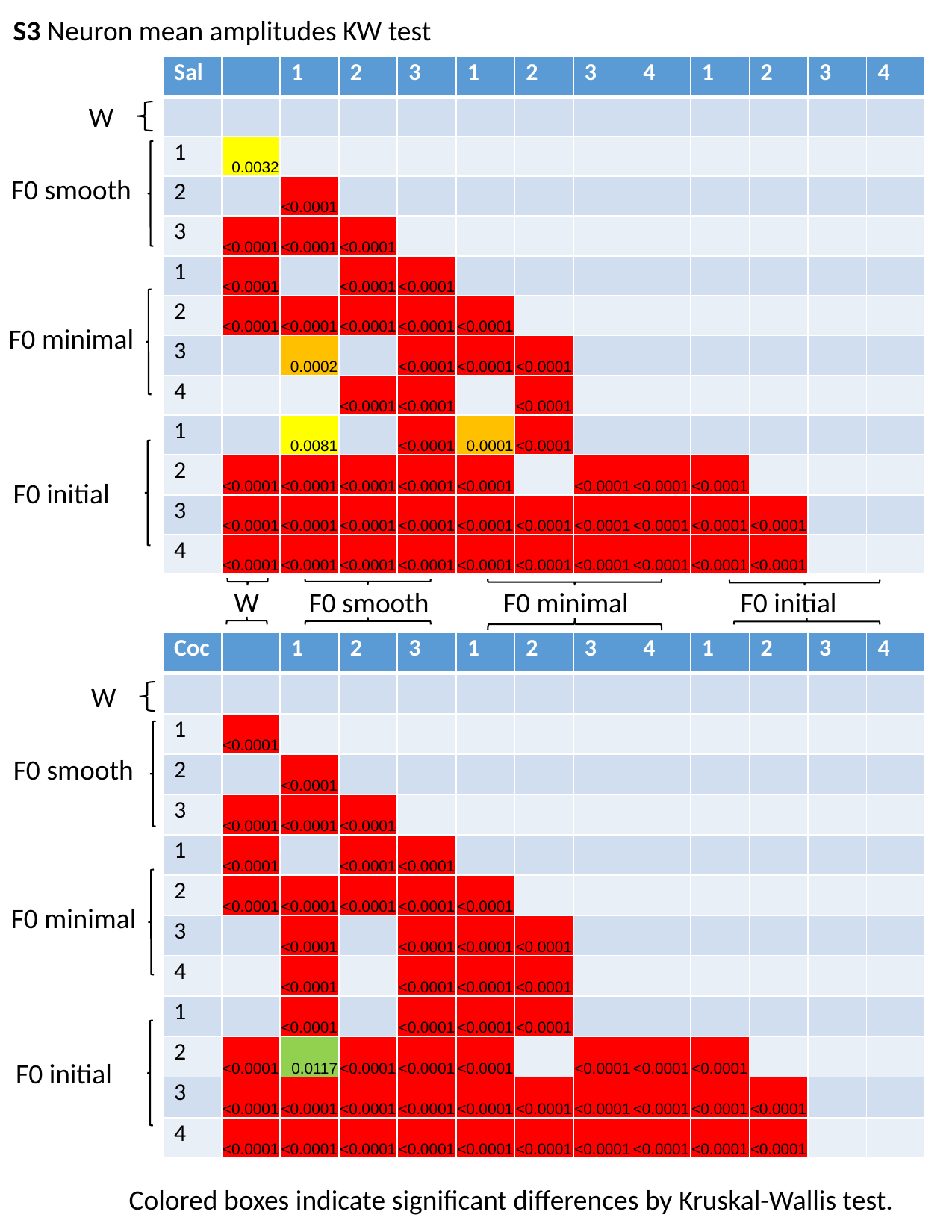

S3 Neuron mean amplitudes KW test
| Sal | | 1 | 2 | 3 | 1 | 2 | 3 | 4 | 1 | 2 | 3 | 4 |
| --- | --- | --- | --- | --- | --- | --- | --- | --- | --- | --- | --- | --- |
| | | | | | | | | | | | | |
| 1 | 0.0032 | | | | | | | | | | | |
| 2 | | <0.0001 | | | | | | | | | | |
| 3 | <0.0001 | <0.0001 | <0.0001 | | | | | | | | | |
| 1 | <0.0001 | | <0.0001 | <0.0001 | | | | | | | | |
| 2 | <0.0001 | <0.0001 | <0.0001 | <0.0001 | <0.0001 | | | | | | | |
| 3 | | 0.0002 | | <0.0001 | <0.0001 | <0.0001 | | | | | | |
| 4 | | | <0.0001 | <0.0001 | | <0.0001 | | | | | | |
| 1 | | 0.0081 | | <0.0001 | 0.0001 | <0.0001 | | | | | | |
| 2 | <0.0001 | <0.0001 | <0.0001 | <0.0001 | <0.0001 | | <0.0001 | <0.0001 | <0.0001 | | | |
| 3 | <0.0001 | <0.0001 | <0.0001 | <0.0001 | <0.0001 | <0.0001 | <0.0001 | <0.0001 | <0.0001 | <0.0001 | | |
| 4 | <0.0001 | <0.0001 | <0.0001 | <0.0001 | <0.0001 | <0.0001 | <0.0001 | <0.0001 | <0.0001 | <0.0001 | | |
W
F0 smooth
F0 minimal
F0 initial
W
F0 smooth
F0 minimal
F0 initial
| Coc | | 1 | 2 | 3 | 1 | 2 | 3 | 4 | 1 | 2 | 3 | 4 |
| --- | --- | --- | --- | --- | --- | --- | --- | --- | --- | --- | --- | --- |
| | | | | | | | | | | | | |
| 1 | <0.0001 | | | | | | | | | | | |
| 2 | | <0.0001 | | | | | | | | | | |
| 3 | <0.0001 | <0.0001 | <0.0001 | | | | | | | | | |
| 1 | <0.0001 | | <0.0001 | <0.0001 | | | | | | | | |
| 2 | <0.0001 | <0.0001 | <0.0001 | <0.0001 | <0.0001 | | | | | | | |
| 3 | | <0.0001 | | <0.0001 | <0.0001 | <0.0001 | | | | | | |
| 4 | | <0.0001 | | <0.0001 | <0.0001 | <0.0001 | | | | | | |
| 1 | | <0.0001 | | <0.0001 | <0.0001 | <0.0001 | | | | | | |
| 2 | <0.0001 | 0.0117 | <0.0001 | <0.0001 | <0.0001 | | <0.0001 | <0.0001 | <0.0001 | | | |
| 3 | <0.0001 | <0.0001 | <0.0001 | <0.0001 | <0.0001 | <0.0001 | <0.0001 | <0.0001 | <0.0001 | <0.0001 | | |
| 4 | <0.0001 | <0.0001 | <0.0001 | <0.0001 | <0.0001 | <0.0001 | <0.0001 | <0.0001 | <0.0001 | <0.0001 | | |
W
F0 smooth
F0 minimal
F0 initial
Colored boxes indicate significant differences by Kruskal-Wallis test.

## Slide 4
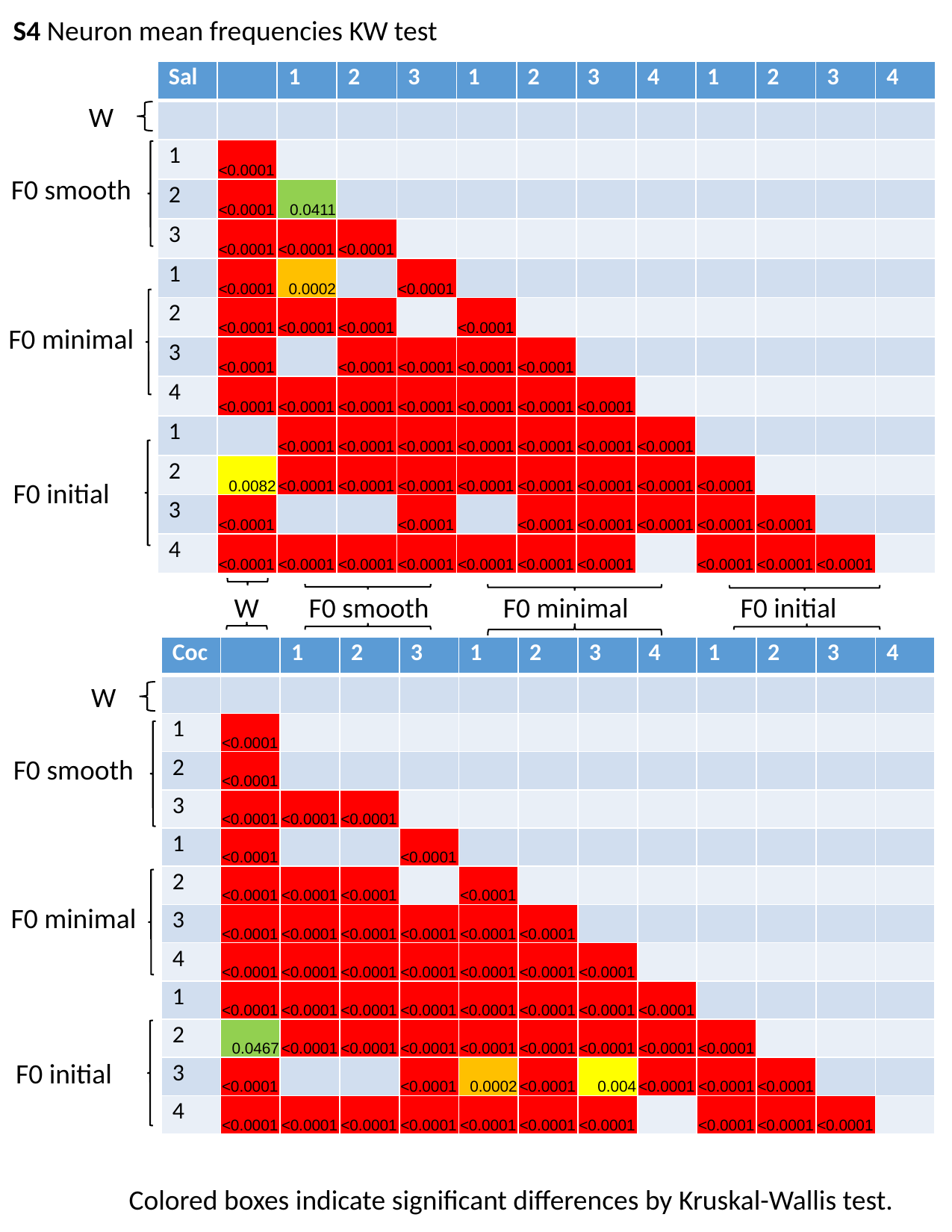

S4 Neuron mean frequencies KW test
| Sal | | 1 | 2 | 3 | 1 | 2 | 3 | 4 | 1 | 2 | 3 | 4 |
| --- | --- | --- | --- | --- | --- | --- | --- | --- | --- | --- | --- | --- |
| | | | | | | | | | | | | |
| 1 | <0.0001 | | | | | | | | | | | |
| 2 | <0.0001 | 0.0411 | | | | | | | | | | |
| 3 | <0.0001 | <0.0001 | <0.0001 | | | | | | | | | |
| 1 | <0.0001 | 0.0002 | | <0.0001 | | | | | | | | |
| 2 | <0.0001 | <0.0001 | <0.0001 | | <0.0001 | | | | | | | |
| 3 | <0.0001 | | <0.0001 | <0.0001 | <0.0001 | <0.0001 | | | | | | |
| 4 | <0.0001 | <0.0001 | <0.0001 | <0.0001 | <0.0001 | <0.0001 | <0.0001 | | | | | |
| 1 | | <0.0001 | <0.0001 | <0.0001 | <0.0001 | <0.0001 | <0.0001 | <0.0001 | | | | |
| 2 | 0.0082 | <0.0001 | <0.0001 | <0.0001 | <0.0001 | <0.0001 | <0.0001 | <0.0001 | <0.0001 | | | |
| 3 | <0.0001 | | | <0.0001 | | <0.0001 | <0.0001 | <0.0001 | <0.0001 | <0.0001 | | |
| 4 | <0.0001 | <0.0001 | <0.0001 | <0.0001 | <0.0001 | <0.0001 | <0.0001 | | <0.0001 | <0.0001 | <0.0001 | |
W
F0 smooth
F0 minimal
F0 initial
W
F0 smooth
F0 minimal
F0 initial
| Coc | | 1 | 2 | 3 | 1 | 2 | 3 | 4 | 1 | 2 | 3 | 4 |
| --- | --- | --- | --- | --- | --- | --- | --- | --- | --- | --- | --- | --- |
| | | | | | | | | | | | | |
| 1 | <0.0001 | | | | | | | | | | | |
| 2 | <0.0001 | | | | | | | | | | | |
| 3 | <0.0001 | <0.0001 | <0.0001 | | | | | | | | | |
| 1 | <0.0001 | | | <0.0001 | | | | | | | | |
| 2 | <0.0001 | <0.0001 | <0.0001 | | <0.0001 | | | | | | | |
| 3 | <0.0001 | <0.0001 | <0.0001 | <0.0001 | <0.0001 | <0.0001 | | | | | | |
| 4 | <0.0001 | <0.0001 | <0.0001 | <0.0001 | <0.0001 | <0.0001 | <0.0001 | | | | | |
| 1 | <0.0001 | <0.0001 | <0.0001 | <0.0001 | <0.0001 | <0.0001 | <0.0001 | <0.0001 | | | | |
| 2 | 0.0467 | <0.0001 | <0.0001 | <0.0001 | <0.0001 | <0.0001 | <0.0001 | <0.0001 | <0.0001 | | | |
| 3 | <0.0001 | | | <0.0001 | 0.0002 | <0.0001 | 0.004 | <0.0001 | <0.0001 | <0.0001 | | |
| 4 | <0.0001 | <0.0001 | <0.0001 | <0.0001 | <0.0001 | <0.0001 | <0.0001 | | <0.0001 | <0.0001 | <0.0001 | |
W
F0 smooth
F0 minimal
F0 initial
Colored boxes indicate significant differences by Kruskal-Wallis test.

## Slide 5
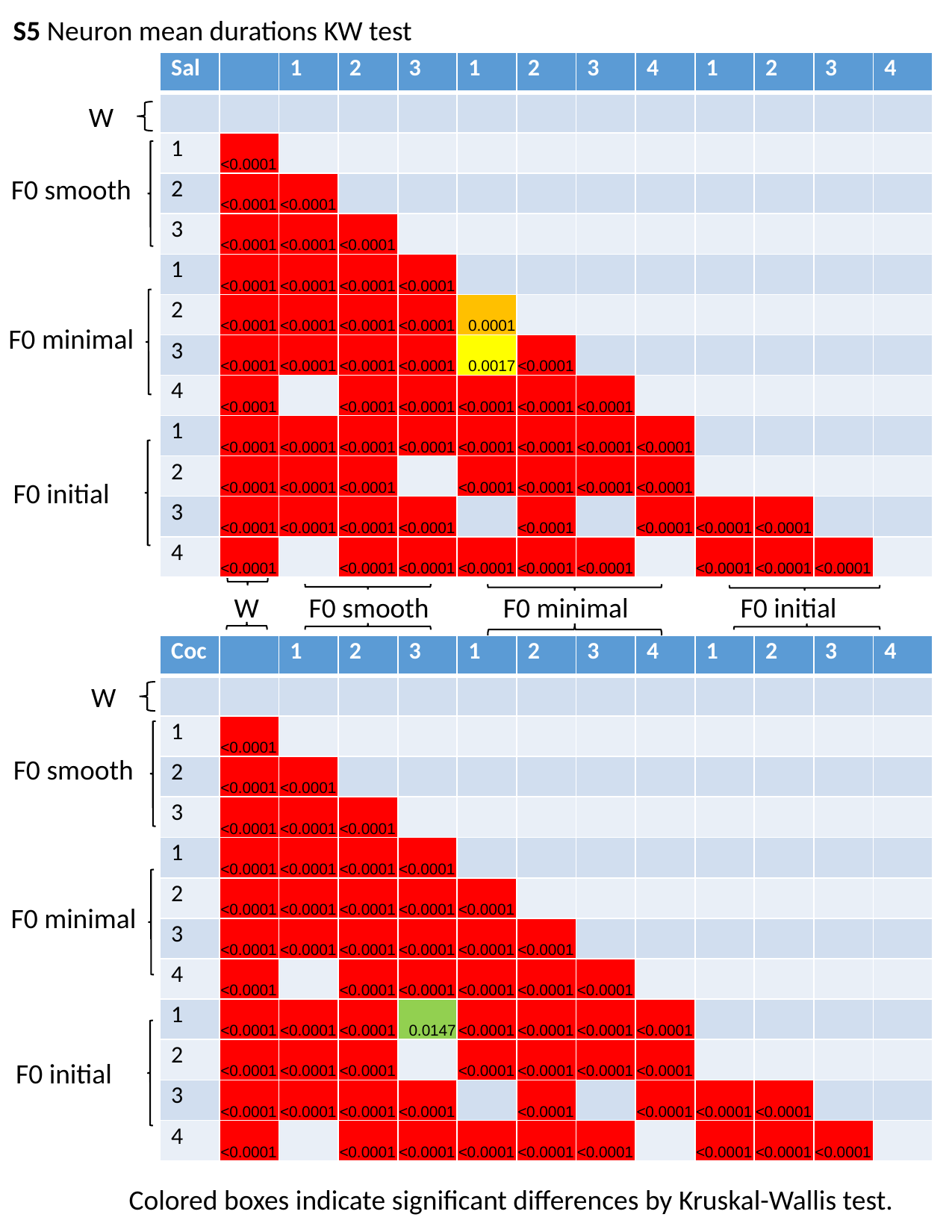

S5 Neuron mean durations KW test
| Sal | | 1 | 2 | 3 | 1 | 2 | 3 | 4 | 1 | 2 | 3 | 4 |
| --- | --- | --- | --- | --- | --- | --- | --- | --- | --- | --- | --- | --- |
| | | | | | | | | | | | | |
| 1 | <0.0001 | | | | | | | | | | | |
| 2 | <0.0001 | <0.0001 | | | | | | | | | | |
| 3 | <0.0001 | <0.0001 | <0.0001 | | | | | | | | | |
| 1 | <0.0001 | <0.0001 | <0.0001 | <0.0001 | | | | | | | | |
| 2 | <0.0001 | <0.0001 | <0.0001 | <0.0001 | 0.0001 | | | | | | | |
| 3 | <0.0001 | <0.0001 | <0.0001 | <0.0001 | 0.0017 | <0.0001 | | | | | | |
| 4 | <0.0001 | | <0.0001 | <0.0001 | <0.0001 | <0.0001 | <0.0001 | | | | | |
| 1 | <0.0001 | <0.0001 | <0.0001 | <0.0001 | <0.0001 | <0.0001 | <0.0001 | <0.0001 | | | | |
| 2 | <0.0001 | <0.0001 | <0.0001 | | <0.0001 | <0.0001 | <0.0001 | <0.0001 | | | | |
| 3 | <0.0001 | <0.0001 | <0.0001 | <0.0001 | | <0.0001 | | <0.0001 | <0.0001 | <0.0001 | | |
| 4 | <0.0001 | | <0.0001 | <0.0001 | <0.0001 | <0.0001 | <0.0001 | | <0.0001 | <0.0001 | <0.0001 | |
W
F0 smooth
F0 minimal
F0 initial
W
F0 smooth
F0 minimal
F0 initial
| Coc | | 1 | 2 | 3 | 1 | 2 | 3 | 4 | 1 | 2 | 3 | 4 |
| --- | --- | --- | --- | --- | --- | --- | --- | --- | --- | --- | --- | --- |
| | | | | | | | | | | | | |
| 1 | <0.0001 | | | | | | | | | | | |
| 2 | <0.0001 | <0.0001 | | | | | | | | | | |
| 3 | <0.0001 | <0.0001 | <0.0001 | | | | | | | | | |
| 1 | <0.0001 | <0.0001 | <0.0001 | <0.0001 | | | | | | | | |
| 2 | <0.0001 | <0.0001 | <0.0001 | <0.0001 | <0.0001 | | | | | | | |
| 3 | <0.0001 | <0.0001 | <0.0001 | <0.0001 | <0.0001 | <0.0001 | | | | | | |
| 4 | <0.0001 | | <0.0001 | <0.0001 | <0.0001 | <0.0001 | <0.0001 | | | | | |
| 1 | <0.0001 | <0.0001 | <0.0001 | 0.0147 | <0.0001 | <0.0001 | <0.0001 | <0.0001 | | | | |
| 2 | <0.0001 | <0.0001 | <0.0001 | | <0.0001 | <0.0001 | <0.0001 | <0.0001 | | | | |
| 3 | <0.0001 | <0.0001 | <0.0001 | <0.0001 | | <0.0001 | | <0.0001 | <0.0001 | <0.0001 | | |
| 4 | <0.0001 | | <0.0001 | <0.0001 | <0.0001 | <0.0001 | <0.0001 | | <0.0001 | <0.0001 | <0.0001 | |
W
F0 smooth
F0 minimal
F0 initial
Colored boxes indicate significant differences by Kruskal-Wallis test.

## Slide 6
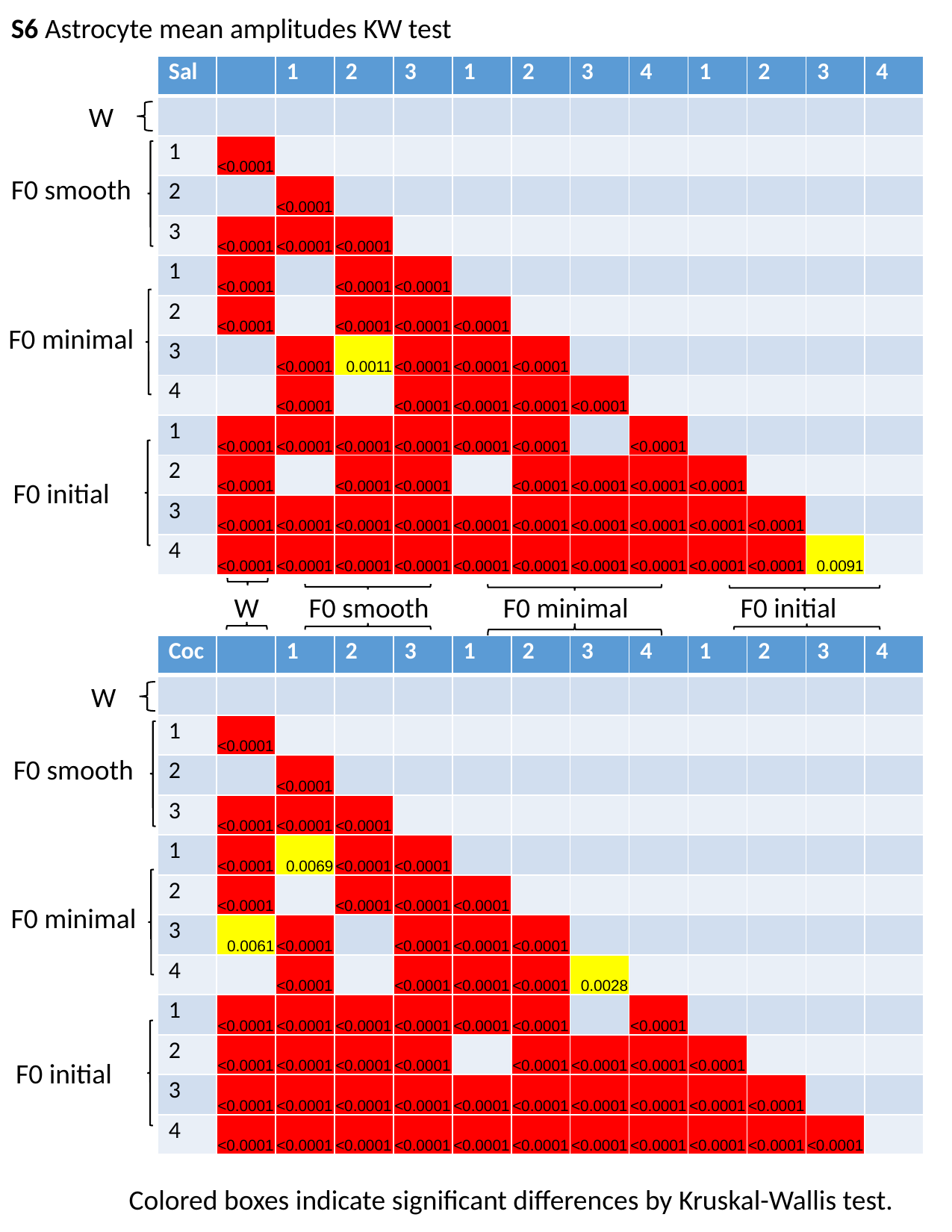

S6 Astrocyte mean amplitudes KW test
| Sal | | 1 | 2 | 3 | 1 | 2 | 3 | 4 | 1 | 2 | 3 | 4 |
| --- | --- | --- | --- | --- | --- | --- | --- | --- | --- | --- | --- | --- |
| | | | | | | | | | | | | |
| 1 | <0.0001 | | | | | | | | | | | |
| 2 | | <0.0001 | | | | | | | | | | |
| 3 | <0.0001 | <0.0001 | <0.0001 | | | | | | | | | |
| 1 | <0.0001 | | <0.0001 | <0.0001 | | | | | | | | |
| 2 | <0.0001 | | <0.0001 | <0.0001 | <0.0001 | | | | | | | |
| 3 | | <0.0001 | 0.0011 | <0.0001 | <0.0001 | <0.0001 | | | | | | |
| 4 | | <0.0001 | | <0.0001 | <0.0001 | <0.0001 | <0.0001 | | | | | |
| 1 | <0.0001 | <0.0001 | <0.0001 | <0.0001 | <0.0001 | <0.0001 | | <0.0001 | | | | |
| 2 | <0.0001 | | <0.0001 | <0.0001 | | <0.0001 | <0.0001 | <0.0001 | <0.0001 | | | |
| 3 | <0.0001 | <0.0001 | <0.0001 | <0.0001 | <0.0001 | <0.0001 | <0.0001 | <0.0001 | <0.0001 | <0.0001 | | |
| 4 | <0.0001 | <0.0001 | <0.0001 | <0.0001 | <0.0001 | <0.0001 | <0.0001 | <0.0001 | <0.0001 | <0.0001 | 0.0091 | |
W
F0 smooth
F0 minimal
F0 initial
W
F0 smooth
F0 minimal
F0 initial
| Coc | | 1 | 2 | 3 | 1 | 2 | 3 | 4 | 1 | 2 | 3 | 4 |
| --- | --- | --- | --- | --- | --- | --- | --- | --- | --- | --- | --- | --- |
| | | | | | | | | | | | | |
| 1 | <0.0001 | | | | | | | | | | | |
| 2 | | <0.0001 | | | | | | | | | | |
| 3 | <0.0001 | <0.0001 | <0.0001 | | | | | | | | | |
| 1 | <0.0001 | 0.0069 | <0.0001 | <0.0001 | | | | | | | | |
| 2 | <0.0001 | | <0.0001 | <0.0001 | <0.0001 | | | | | | | |
| 3 | 0.0061 | <0.0001 | | <0.0001 | <0.0001 | <0.0001 | | | | | | |
| 4 | | <0.0001 | | <0.0001 | <0.0001 | <0.0001 | 0.0028 | | | | | |
| 1 | <0.0001 | <0.0001 | <0.0001 | <0.0001 | <0.0001 | <0.0001 | | <0.0001 | | | | |
| 2 | <0.0001 | <0.0001 | <0.0001 | <0.0001 | | <0.0001 | <0.0001 | <0.0001 | <0.0001 | | | |
| 3 | <0.0001 | <0.0001 | <0.0001 | <0.0001 | <0.0001 | <0.0001 | <0.0001 | <0.0001 | <0.0001 | <0.0001 | | |
| 4 | <0.0001 | <0.0001 | <0.0001 | <0.0001 | <0.0001 | <0.0001 | <0.0001 | <0.0001 | <0.0001 | <0.0001 | <0.0001 | |
W
F0 smooth
F0 minimal
F0 initial
Colored boxes indicate significant differences by Kruskal-Wallis test.

## Slide 7
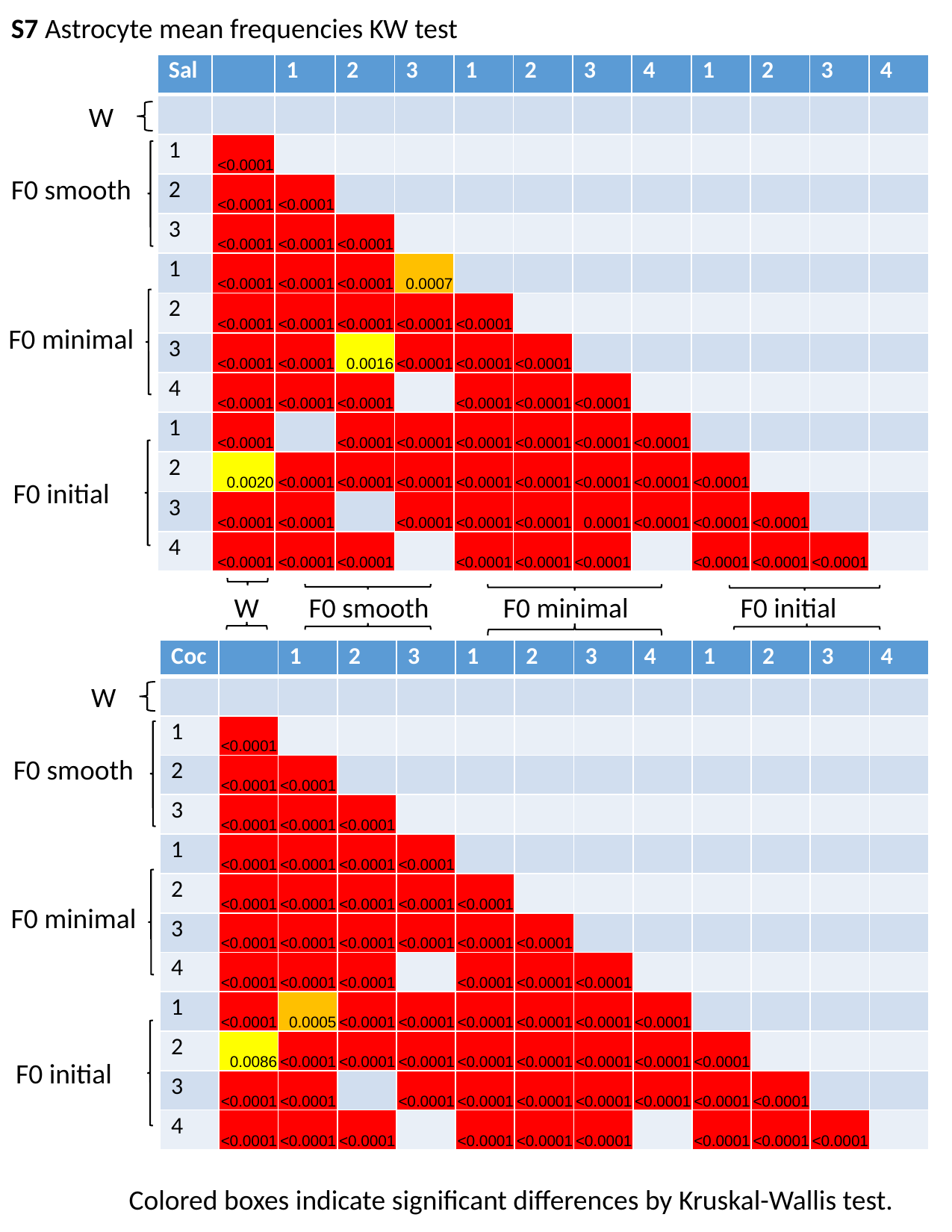

S7 Astrocyte mean frequencies KW test
| Sal | | 1 | 2 | 3 | 1 | 2 | 3 | 4 | 1 | 2 | 3 | 4 |
| --- | --- | --- | --- | --- | --- | --- | --- | --- | --- | --- | --- | --- |
| | | | | | | | | | | | | |
| 1 | <0.0001 | | | | | | | | | | | |
| 2 | <0.0001 | <0.0001 | | | | | | | | | | |
| 3 | <0.0001 | <0.0001 | <0.0001 | | | | | | | | | |
| 1 | <0.0001 | <0.0001 | <0.0001 | 0.0007 | | | | | | | | |
| 2 | <0.0001 | <0.0001 | <0.0001 | <0.0001 | <0.0001 | | | | | | | |
| 3 | <0.0001 | <0.0001 | 0.0016 | <0.0001 | <0.0001 | <0.0001 | | | | | | |
| 4 | <0.0001 | <0.0001 | <0.0001 | | <0.0001 | <0.0001 | <0.0001 | | | | | |
| 1 | <0.0001 | | <0.0001 | <0.0001 | <0.0001 | <0.0001 | <0.0001 | <0.0001 | | | | |
| 2 | 0.0020 | <0.0001 | <0.0001 | <0.0001 | <0.0001 | <0.0001 | <0.0001 | <0.0001 | <0.0001 | | | |
| 3 | <0.0001 | <0.0001 | | <0.0001 | <0.0001 | <0.0001 | 0.0001 | <0.0001 | <0.0001 | <0.0001 | | |
| 4 | <0.0001 | <0.0001 | <0.0001 | | <0.0001 | <0.0001 | <0.0001 | | <0.0001 | <0.0001 | <0.0001 | |
W
F0 smooth
F0 minimal
F0 initial
W
F0 smooth
F0 minimal
F0 initial
| Coc | | 1 | 2 | 3 | 1 | 2 | 3 | 4 | 1 | 2 | 3 | 4 |
| --- | --- | --- | --- | --- | --- | --- | --- | --- | --- | --- | --- | --- |
| | | | | | | | | | | | | |
| 1 | <0.0001 | | | | | | | | | | | |
| 2 | <0.0001 | <0.0001 | | | | | | | | | | |
| 3 | <0.0001 | <0.0001 | <0.0001 | | | | | | | | | |
| 1 | <0.0001 | <0.0001 | <0.0001 | <0.0001 | | | | | | | | |
| 2 | <0.0001 | <0.0001 | <0.0001 | <0.0001 | <0.0001 | | | | | | | |
| 3 | <0.0001 | <0.0001 | <0.0001 | <0.0001 | <0.0001 | <0.0001 | | | | | | |
| 4 | <0.0001 | <0.0001 | <0.0001 | | <0.0001 | <0.0001 | <0.0001 | | | | | |
| 1 | <0.0001 | 0.0005 | <0.0001 | <0.0001 | <0.0001 | <0.0001 | <0.0001 | <0.0001 | | | | |
| 2 | 0.0086 | <0.0001 | <0.0001 | <0.0001 | <0.0001 | <0.0001 | <0.0001 | <0.0001 | <0.0001 | | | |
| 3 | <0.0001 | <0.0001 | | <0.0001 | <0.0001 | <0.0001 | <0.0001 | <0.0001 | <0.0001 | <0.0001 | | |
| 4 | <0.0001 | <0.0001 | <0.0001 | | <0.0001 | <0.0001 | <0.0001 | | <0.0001 | <0.0001 | <0.0001 | |
W
F0 smooth
F0 minimal
F0 initial
Colored boxes indicate significant differences by Kruskal-Wallis test.

## Slide 8
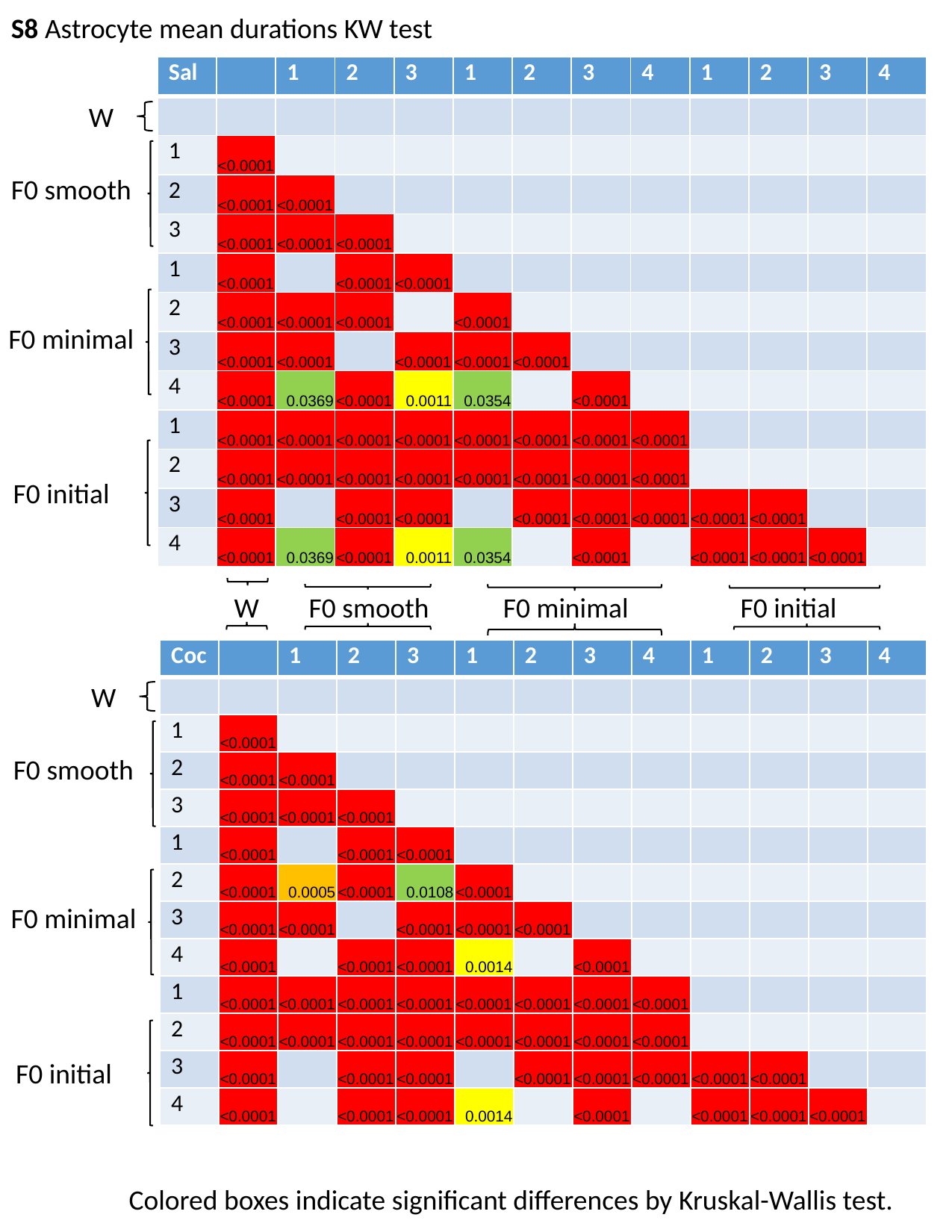

S8 Astrocyte mean durations KW test
| Sal | | 1 | 2 | 3 | 1 | 2 | 3 | 4 | 1 | 2 | 3 | 4 |
| --- | --- | --- | --- | --- | --- | --- | --- | --- | --- | --- | --- | --- |
| | | | | | | | | | | | | |
| 1 | <0.0001 | | | | | | | | | | | |
| 2 | <0.0001 | <0.0001 | | | | | | | | | | |
| 3 | <0.0001 | <0.0001 | <0.0001 | | | | | | | | | |
| 1 | <0.0001 | | <0.0001 | <0.0001 | | | | | | | | |
| 2 | <0.0001 | <0.0001 | <0.0001 | | <0.0001 | | | | | | | |
| 3 | <0.0001 | <0.0001 | | <0.0001 | <0.0001 | <0.0001 | | | | | | |
| 4 | <0.0001 | 0.0369 | <0.0001 | 0.0011 | 0.0354 | | <0.0001 | | | | | |
| 1 | <0.0001 | <0.0001 | <0.0001 | <0.0001 | <0.0001 | <0.0001 | <0.0001 | <0.0001 | | | | |
| 2 | <0.0001 | <0.0001 | <0.0001 | <0.0001 | <0.0001 | <0.0001 | <0.0001 | <0.0001 | | | | |
| 3 | <0.0001 | | <0.0001 | <0.0001 | | <0.0001 | <0.0001 | <0.0001 | <0.0001 | <0.0001 | | |
| 4 | <0.0001 | 0.0369 | <0.0001 | 0.0011 | 0.0354 | | <0.0001 | | <0.0001 | <0.0001 | <0.0001 | |
W
F0 smooth
F0 minimal
F0 initial
W
F0 smooth
F0 minimal
F0 initial
| Coc | | 1 | 2 | 3 | 1 | 2 | 3 | 4 | 1 | 2 | 3 | 4 |
| --- | --- | --- | --- | --- | --- | --- | --- | --- | --- | --- | --- | --- |
| | | | | | | | | | | | | |
| 1 | <0.0001 | | | | | | | | | | | |
| 2 | <0.0001 | <0.0001 | | | | | | | | | | |
| 3 | <0.0001 | <0.0001 | <0.0001 | | | | | | | | | |
| 1 | <0.0001 | | <0.0001 | <0.0001 | | | | | | | | |
| 2 | <0.0001 | 0.0005 | <0.0001 | 0.0108 | <0.0001 | | | | | | | |
| 3 | <0.0001 | <0.0001 | | <0.0001 | <0.0001 | <0.0001 | | | | | | |
| 4 | <0.0001 | | <0.0001 | <0.0001 | 0.0014 | | <0.0001 | | | | | |
| 1 | <0.0001 | <0.0001 | <0.0001 | <0.0001 | <0.0001 | <0.0001 | <0.0001 | <0.0001 | | | | |
| 2 | <0.0001 | <0.0001 | <0.0001 | <0.0001 | <0.0001 | <0.0001 | <0.0001 | <0.0001 | | | | |
| 3 | <0.0001 | | <0.0001 | <0.0001 | | <0.0001 | <0.0001 | <0.0001 | <0.0001 | <0.0001 | | |
| 4 | <0.0001 | | <0.0001 | <0.0001 | 0.0014 | | <0.0001 | | <0.0001 | <0.0001 | <0.0001 | |
W
F0 smooth
F0 minimal
F0 initial
Colored boxes indicate significant differences by Kruskal-Wallis test.
